# Uncovering the cognitive mechanisms underlying the gaze cueing effect

**DOI:** 10.1177/17470218231181238

**Published:** 2023-06-27

**Authors:** Manikya Alister, Kate T McKay, David K Sewell, Nathan J Evans

**Affiliations:** 1 Melbourne School of Psychological Sciences, The University of Melbourne, Melbourne, VIC, Australia; 2 School of Psychology, The University of Queensland, Saint Lucia, QLD, Australia; 3 Department of Psychology, Ludwig Maximilian University of Munich, Munich, Germany

**Keywords:** Gaze, cueing, social cognition, computational modelling, attention, decision-making

## Abstract

The gaze cueing effect is the tendency for people to respond faster to targets appearing at locations gazed at by others, compared with locations gazed away from by others. The effect is robust, widely studied, and is an influential finding within social cognition. Formal evidence accumulation models provide the dominant theoretical account of the cognitive processes underlying speeded decision-making, but they have rarely been applied to social cognition research. In this study, using a combination of individual-level and hierarchical computational modelling techniques, we applied evidence accumulation models to gaze cueing data (three data sets total, *N* = 171, 139,001 trials) for the first time to assess the relative capacity that an attentional orienting mechanism and information processing mechanisms have for explaining the gaze cueing effect. We found that most participants were best described by the attentional orienting mechanism, such that response times were slower at gazed away from locations because they had to reorient to the target before they could process the cue. However, we found evidence for individual differences, whereby the models suggested that some gaze cueing effects were driven by a short allocation of information processing resources to the gazed at location, allowing for a brief period where orienting and processing could occur in parallel. There was exceptionally little evidence to suggest any sustained reallocation of information processing resources neither at the group nor individual level. We discuss how this individual variability might represent credible individual differences in the cognitive mechanisms that subserve behaviourally observed gaze cueing effects.

Eyes reveal rich social information about the intentions, emotional states, and desires of others (Stephenson et al., 2021). Therefore, being attentive to the direction of other people’s eye gaze is an important social-cognitive capacity, reflected by a robust tendency for people to respond faster to locations gazed-at by others compared with locations gazed away from by others (Frischen et al., 2007; McKay et al., 2021). This tendency is referred to as the gaze cueing effect (as initially demonstrated in Driver et al., 1999 and Friesen & Kingstone, 1998).

The gaze cueing effect is a particularly interesting phenomenon, because following the direction of other people’s gaze is considered to be critical in the development of human social cognition (see Argyle & Cook, 1976; Baron-Cohen & Belmonte, 2005; Emery, 2000; Kleinke, 1986; Renfrew et al., 2008; and Stephenson et al., 2021 for theoretical reviews). This interest has been reflected in the published literature, with the gaze cueing effect being studied widely and across a range of contexts. A recent meta-analysis of more than 400 gaze cueing effects found that it is remarkably robust, emerging irrespective of a number of task- and cue-feature parameters in healthy adults (see McKay et al., 2021). However, despite the gaze cueing effect being studied extensively, there is still little known about the specific cognitive mechanisms involved in or necessary for its emergence.

One way in which the nature of these faster response times at gaze cued rather than gaze miscued locations can be better understood is through mathematical “process models,” also referred to as “computational models” of human cognition. These models are a causal theoretical explanation for how a “cognitive” phenomenon, like decision-making, occurs, rather than simply a mathematical summary of a functional form, such as curve fitting. Computational models attempt to describe mechanisms underlying cognitive processes by constructing mathematically defined theoretical predictions with clearly identified assumptions (see Lewandowsky & Farrell, 2011). These models can then be applied to empirical datasets in order to test and compare the efficacy of these predictions and the utility of different models’ assumptions for explaining the given behaviour. Thus, these computational models hold many advantages over traditional statistical analyses of observable behavioural data alone because these models directly map on to theories of cognitive processes and their predictions can be directly assessed against observed data (for more detail on the advantages of a computational modelling approach, see Guest & Martin, 2021). These features of computational models are also useful for discerning whether two ostensibly equivalent patterns of empirical data can be understood in terms of a common psychological mechanism, or whether they require different explanations (e.g., Imhoff et al., 2019).

Evidence accumulation models are a class of computational models that jointly address response time and accuracy data to infer several cognitive mechanisms underlying a decision, and have become the dominant theoretical framework for describing speeded decisions (see Evans & Wagenmakers, 2019; Ratcliff et al., 2016 for reviews). However, although the gaze cueing effect arises from data derived from a speeded decision task, it is still unclear how evidence accumulation mechanisms contribute to the emergence of the gaze cueing effect, despite recent calls for their wider use in social cognition research (Parker & Ramsey, 2022b). Indeed, there has only been one other study that has attempted to model the gaze cueing effect using an evidence accumulation framework, and that study only tested one potential underlying mechanism (Parker & Ramsey, 2022a).

In this study then, we investigated multiple possible mechanisms driving the gaze cueing effect by adapting an established and commonly used evidence accumulation model of decision-making, the diffusion decision model (DDM; Ratcliff & McKoon, 2007; Ratcliff & Rouder, 1998; Stone, 1960), and fitting these adaptations to pre-existing data from several gaze cueing studies. In the following sections, we summarise the current understanding of the gaze cueing effect and show how this understanding could be strengthened by investigating the gaze cueing effect within a computational modelling framework. We then describe evidence accumulation models, summarising their impact within cognitive psychology, their properties, and how they may apply to the emergence of the gaze cueing effect.

## The gaze cueing effect

The gaze cueing effect has been studied broadly and has implications for a variety of theoretical frameworks (e.g., see Stephenson et al., 2021) and clinical applications (e.g., Catalano et al., 2020; de Araújo et al., 2020; Langdon et al., 2017; Magnée et al., 2011; Senju et al., 2003). Empirically, the gaze cueing effect has been demonstrated using the gaze cueing paradigm (see Driver et al., 1999; Friesen & Kingstone, 1998, for seminal studies). In this paradigm, participants are presented with a face with averted eyes in the centre of the screen and then a target to be responded to at the peripheral left or right (e.g., the sudden onset of an asterisk; as in Gregory & Jackson, 2020). On cued trials, the target appears at the gazed-at location—that is, the side of the screen that the central faces averted gaze is oriented towards. On miscued trials, the target appears at the gazed-away from location—that is, the side of the screen that the central faces averted gaze is oriented away from. The gaze cueing effect describes the typical finding that faster mean response times arise for cued versus miscued trials.

The gaze cueing effect is commonly characterised as a covert, stimulus-driven, reflexive shift of attention towards the gazed at location via the eye gaze cue. To borrow Posner et al.’s (1980) terminology, this characterisation reflects the attentional orienting capacity of eye gaze. The initial orienting of attention, in turn, enables more detailed processing of stimuli that fall within its focus. Behaviourally, the gaze cueing effect emerges even when the cue is presented after a very brief cue–target stimulus onset asynchrony (SOA; e.g., Friesen & Kingstone, 1998). The effect has been observed when the cue is presented very briefly and then masked (e.g., Sato et al., 2007), when cues are non-predictive (e.g., Galfano et al., 2012), counter predictive (Kuhn & Kingstone, 2009; Tipples, 2008), and even when participants are explicitly told to ignore the cue (e.g., Gregory & Jackson, 2020). Taken together, this suggests that the effect is rapidly, unconsciously, and involuntarily produced. Supporting this, neural evidence has suggested that the gaze cueing effect emerges very early in the attentional processing hierarchy (e.g., Hietanen et al., 2008), activating brain regions associated with pre-conscious, exogenous attention shifts (e.g., Joseph et al., 2015).

In addition to its attentional orienting properties, eye gaze has also been found to influence subsequent information processing. For example, infants tend to process stimuli more efficiently when they are looked at by others, and have better memory for gazed-at targets (Reid et al., 2004; Reid & Striano, 2005; Wahl et al., 2013, 2019), which is consistent with broader theories of joint attention suggesting people allocate more information processing resources to locations that they are aware another person is looking at (Shteynberg, 2015). In addition the reverse is also true. Elements in the environment typically characterised as requiring more information processing, including various aspects of person perception and the relationship between the observer and the gazing person, influence the magnitude of gaze cueing effects (Dalmaso et al., 2020). A recent study applying evidence accumulation models also suggested that the response time boost to gazed at locations could be a result of more efficient information processing at the gazed-at location (Parker & Ramsey, 2022a); however, they *only* considered an information processing mechanism, so could not distinguish between other mechanisms (such as an attentional orienting mechanism) that could potentially be driving the effect. Moreover, despite these studies describing how information processing may be affected by gaze cues, the precise nature of these theorised shifts in information processing efficiency to gazed at locations has not previously been defined. Particularly, whether eye gaze results in a sustained boost in information processing to the gazed at location or whether it is only an initial information processing boost that dissipates or is redistributed across the visual field over time.

In this section, we have outlined different ways that the emergence of the gaze cueing effect has been described in the literature. According to some accounts, gaze cues serve to automatically orient attention to stimuli, speeding the onset of early encoding processes. Other accounts place less emphasis on gaze cues altering the time course of stimulus processing, instead focusing on enhanced processing of encoded information. These competing perspectives align with the different classes of processing mechanisms that have also been discussed in the wider visual attention literature. Sewell and Smith (2012) considered the distinction between *gain* (or *resource allocation*) and *orienting* models of attention. Resource allocation models conceive of attention as a mechanism for distributing a limited capacity of processing resources among stimuli in the environment (Kahneman, 1973). According to these models, the spatiotemporal distribution of processing resources determines the efficiency of stimulus processing, such that processing of attended stimuli is enhanced relative to unattended stimuli, resulting in more robust and higher quality stimulus representations (e.g., Sperling & Weichselgartner, 1995). By contrast, orienting models view attention as a mechanism for selecting stimuli for subsequent processing (Broadbent, 1958). According to these models, attention must first be oriented to the stimulus before processing can begin, an idea perhaps most influentially expressed by Posner et al.’s (1980) spotlight metaphor for attention. In orienting models, attention primarily controls the time course of processing, resulting in more rapid encoding of attended versus unattended stimuli.

To date, there has not yet been a model-based attempt to distinguish between resource allocation and orienting accounts of the gaze cueing effect, or alternatively, to quantify the relative contributions of resource allocation and orienting mechanisms to producing the gaze cueing effect. One approach to this issue is to use evidence accumulation models to decompose the gaze cueing effect into processing components that map onto attention orienting mechanisms that control the time course of processing and resource allocation mechanisms that control the quality of stimulus encoding. In the next section, we elaborate on these different mechanisms described by evidence accumulation models, and propose how these mechanisms could be driving the gaze cueing effect.

## Evidence accumulation models of two-choice decision-making

**Figure 1.**
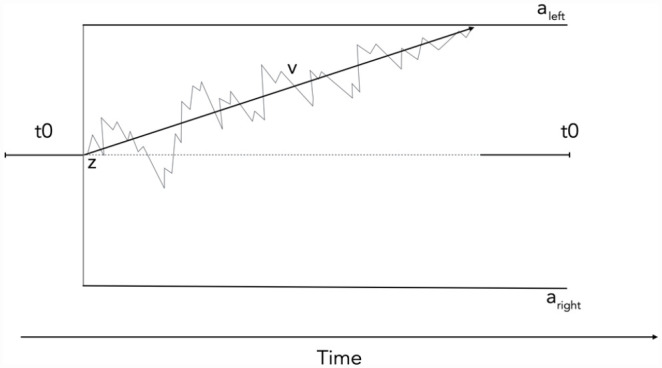
Schematic of a popular evidence accumulation model, the Diffusion Decision Model ratcliff_diffusion_2007 for a single decision. Specifically, it shows noisy evidence accumulation (v) from some starting point (z), until the decision threshold for one of two choices is reached. In the context of the localisation gaze cueing paradigms used in the current study, the two choices could be either the left-sided decision $\left(a_{left}\right)$ or right-sided decision $\left(a_{right}\right)$ . t0 refers to the components unrelated to accumulation over the decision such as perceptual encoding and motor response.

Evidence accumulation models, also known as sequential sampling models (see Donkin & Brown, 2018; Evans & Wagenmakers, 2019; Forstmann et al., 2016; Ratcliff et al., 2016 for reviews), are a well-established class of computational models that provide a widely used, robust theoretical framework from which the cognitive processes underlying speeded decisions can be inferred. According to these models, decision-making is described as the accrual of evidence for (or drift towards) different choices over time at some rate (referred to as the “drift rate”) from some starting point to some decision-triggering threshold. Once that threshold is reached, a response is initiated (see Figure 1 for a visual depiction).

Evidence accumulation models have been used to model decisions across a range of contexts, tasks, and paradigms, and have strengthened our theoretical understanding within a variety of cognitive fields such as memory (e.g., Ratcliff et al., 2004; Zhou et al., 2021), attention (e. g., Hawkins et al., 2019), perception (e.g., Usher & McClelland, 2001; van Ravenzwaaij et al., 2012), decision-making (e.g., Brown & Heathcote, 2008; Ratcliff & McKoon, 2007), intelligence (e.g., Lerche et al., 2020; van Ravenzwaaij et al., 2011), performance optimality (e.g., Evans et al., 2019; Starns & Ratcliff, 2012), emotion processing (e.g., Lerche et al., 2021), and alcohol consumption (e.g., van Ravenzwaaij et al., 2012). In addition, the processes described in these models map onto neural activity observed during decision-making tasks (e.g., Brosnan et al., 2020; Gold & Shadlen, 2007; Liu & Pleskac, 2011; van Maanen et al., 2021), acting as convincing convergent evidence supporting the theoretical assumptions of these models and their explanations of decision-making processes. Given that these models provide the dominant theoretical account of decision-making to date, it is important that we understand the gaze cueing effect through the lens of formal evidence accumulation theories of decision-making.

Using the DDM evidence accumulation model specifically (Ratcliff & Rouder, 1998), in this study we consider three potential components that could plausibly subserve gaze cueing effects: starting point (z), drift rate (v), and/or non-decision time (t0). Each of these three components assumed to be an index of a specific psychological construct that is directly estimated in evidence accumulation models. We describe how each of these components map onto psychological constructs in detail in the following paragraphs and we direct the reader to Figure 1 for a visual representation of each of the model components.

The time course of processes not related to decision-making (i.e., those not involved in the evidence accumulation process) is summarised by the non-decision time parameter. Non-decision time encompasses the necessary early, pre-conscious perceptual encoding of the cue as well as the post-decision motor responses required to behaviourally indicate the decision. In cueing paradigms, we interpret this parameter as being sensitive to attention orienting effects for cued versus miscued trials (Sewell & Smith, 2012; Smith & Ratcliff, 2009), and recognise it is indexing the time course of attentional selection more generally^1^ (Lilburn & Smith, 2020; Sewell et al., 2016; Shepherdson, 2020; Smith et al., 2016). Specifically, a reflexive orienting of attention to the gazed-at location results in the target being processed later in miscued trials compared with cued trials (Sewell & Smith, 2012; Smith & Ratcliff, 2009). The delayed onset of stimulus processing (and therefore decision-making) is naturally reflected in the non-decision time parameter.

As such, a non-decision time interpretation of gaze cueing describes the gaze cueing effect to be caused by orienting costs that are separate from the processing of encoded information. An alternative explanation is that response time differences to cued and miscued targets are driven by increased allocation of information processing resources to the cued location, consistent with resource allocation theories of attention (Kahneman, 1973; Sperling & Weichselgartner, 1995). These information processing mechanisms can be represented by two parameters in the DDM: starting point and drift rate. Typically, the starting point parameter reflects a priori decision-making biases, where the participant is biased towards a particular response alternative before the onset of a specific decision. In gaze cueing tasks with an equal number of cued and miscued trials (i.e., non-predictive tasks, which is what we use in the current study), this typical characterisation of starting point as a response bias is not particularly plausible given that there is no reason for a participant to think that the cue is actually predictive of the target location. A more plausible interpretation of starting point in these contexts involves the presence of more complex processing dynamics than the standard model assumes. If the effect of the cue is to shift a very brief burst of information processing resources to the cued location as the system configures to a regular mode of stimulus processing, the standard model would characterise the evidence accumulated during this initial burst as a shift in the start-point of regular evidence accumulation (e.g., Weigard et al., 2019).^2^ For an unbiased decision, or one with no initial information processing benefit, the starting point of evidence accumulation is situated exactly in the middle of the two response boundaries (as shown in Figure 1). If a person has a initial information processing boost, the starting point of evidence accumulation would be shifted towards the cued response boundary. This means less evidence will be required to trigger the cued response, resulting in faster response times and a greater number of responses relative to the non-favoured response alternative. Importantly, any influence of starting point will exert the same effect regardless of whether the eventual target was cued or miscued, as these processes would be occurring after the appearance of the cue but before the appearance of the target. In terms of the specifications of the model parameters themselves, a key difference between non-decision time and starting point is that the starting point is a function of both response time and accuracy, whereas non-decision time is only a function of response time.

The other parameter that maps onto resource allocation theories of attention in the DDM is drift rate. Drift rate corresponds to the overall quality of evidence driving the evidence accumulation process. Or in other words, the rate of evidence accumulation from the initial appearance of the target until the decision threshold is reached, with a higher drift indicating higher-quality stimulus information and more efficient information processing (see Figure 1; Voss et al., 2004; White & Poldrack, 2014). Importantly, whereas starting point in non-predictive gaze cueing contexts represents an initial boost in information processing to the cued location that dissipates over time, drift rate represents a *sustained* boost in information processing from cue presentation until the response. According to a drift rate account of gaze cueing, information processing resources are preferentially allocated to the cued location, meaning that stimulus information that appears at the cued location (e.g., the target on cued trials) is processed more efficiently compared with stimulus information appearing at the miscued location, resulting in a more robust representation of the stimulus (Sewell & Smith, 2012; Smith & Ratcliff, 2009, consistent with resource allocation models of attention). The only other study to investigate the gaze cueing effect using evidence accumulation models suggested that drift rate underlies response time differences between cued and miscued trials. However, their modelling approach did not allow them to assess the influence of starting point and non-decision time, meaning that their results could just be due to the fact that they did not test any alternative mechanisms (Parker & Ramsey, 2022a). In relation to resource allocation theories of attention, drift rate and starting point vary in the way that that information processing resources are allocated across the duration of the trial. While both drift rate and starting point predict a boost of information processing to the cued location, starting point predicts that this boost only happens initially, after which these resources are gradually redistributed across the visual field.

This capacity for evidence accumulation models to differentiate between underlying cognitive mechanisms is what makes them valuable for understanding gaze cueing effects. Another advantage of these models is that they can make these inferences at the level of individual participants, which is helpful because much of gaze cueing research has focused on identifying differences in the magnitude of gaze cueing between different populations, such as younger versus older people (e.g., for a review see McKay et al., 2022) or autistic people versus control groups (e.g., Sato et al., 2010; Senju et al., 2003), yet these studies predominantly assess for mean group level differences rather than differences across individuals. This is limiting given the considerable heterogeneity that exists in whether and to what extent specific cognitive abilities are affected by ageing, autism, and other populations of interest (Lord et al., 2018; Salthouse, 2010).

In the current study, we use evidence accumulation models to answer a foundational question about the emergence of the gaze cueing effect. Namely, what is the relative contribution of attentional orienting mechanisms (orienting account of attention; Broadbent, 1958) versus resource allocation mechanisms (resource allocation account of attention; Kahneman, 1973; Sperling & Weichselgartner, 1995)? We looked at whether three specific cognitive mechanisms described by evidence accumulation models could explain differences in behaviour across cued and miscued trials: non-decision time, starting point, and drift rate. This was done by fitting variants of the simple DDM (Stone, 1960), which does not include between trial variability in any parameters,^3^ to data from three published experiments by Carlson (2016), Chen et al. (2021), and Gregory and Jackson (2020) using individual-level and group-level analysis techniques.

## Method

### Preregistration and data availability

The analyses for Data Sets 2 (osf.io/5bd8n/?view_only=da1caa2913a4703bc92fa8637205e47) and 3 (osf.io/8sfap/?view_only=55524b2930364fa28a01cebe91380dee) were preregistered after analysing Data Set 1. We followed the preregistration template for the application of computational models by Cruwell and Evans (2019). Any deviations from the preregistered plan are discussed within the manuscript. All of the data and analysis scripts used for this study are available on the github: https://github.com/ManikyaAlister/Gaze-Cueing. Data Sets 1 (osf.io/g62yu/) and 3 (osf.io/6sd4f/?view_only=70bdebcbc248462486ec2fe4eaa2ed07) had already been made available by the original authors, and Data Set 2 was made available at the above OSF address with permission from the original author.

### Data sets

We were able to access the raw data of three studies that applied cueing paradigms appropriate for use in the current study: Gregory and Jackson (2020), Carlson (2016), and Chen et al. (2021) (see Table 1). All of the data used in this study were derived from non-predictive localisation cueing paradigms. That is, targets were cued or miscued by the centrally presented gaze cue an equal number of times and participants had to decide whether the target appeared either at the left or at the right hand side of the screen (more detail on each specific experiment is provided in the following sections). While there are several types of cueing paradigms commonly used in the literature such as categorisation, detection, and localisation paradigms, in this study we only used localisation cueing paradigms in order to facilitate comparison across data sets. These tasks are also the most commonly used in healthy adult populations, according to a recent meta analysis that found almost half of the gaze cueing effects in the published literature are derived from localisation paradigms (McKay et al., 2021). Our method for selecting our data sets was as follows. We searched open data repositories such as the Open Science Framework for localisation gaze cueing paradigms with a sufficient number of trials to be modelled by the DDM (a minimum of 100 trials per cue condition), which yielded two data sets (Data Sets 1 and 3). We also contacted the authors of several studies that had suitable paradigms, which is how we obtained Data Set 2.

**Table 1.** Summary of data sets.

Data set	Original study	*n*	Trials analysed	Mean age	Location
1	Gregory & Jackson, 2020	41	9,826	21	In lab
2	Carlson, 2016	50	12,786	20.6	In lab
3	Chen et al., 2021	71	106,570	19.8	In lab

Location refers to the testing location (i.e., online or in the lab).

To maintain consistency, we applied the same exclusion criteria to each data set. Any trials where the response time was less than 100 ms or greater than 5,000 ms were excluded (these criteria are more liberal than was used in Carlson, 2016, who excluded trials with response times <150 and >750 ms). Furthermore, any participants with less than 80% accuracy were excluded (as is common practice in gaze cueing experiments, see Chen et al., 2021; Coy et al., 2018).

It is important to note that we ignored the distinction between several potential moderating factors in the data sets used, such as cue emotional expression and stimulus onset asynchrony. The main reason we did this was because in most of the data sets there were not enough trials per condition to model these moderating factors (except for Data Set 3 where we do analyse some potential moderating factors in Supplementary Materials 14 and 15, and conclude that these factors did not affect our main findings). Furthermore, ignoring these distinctions facilitated comparison across data sets because these potential moderating factors were not always manipulated the same way across data sets. In the following paragraphs, we briefly describe each experiment that we extracted data from, but refer the reader to the original studies for more information on the original study methods. In these paragraphs we also provide more detail on the potential moderating factors in each of the data sets.

#### Data Set 1: Gregory and Jackson (2020) Experiment 1

**Figure 2.**
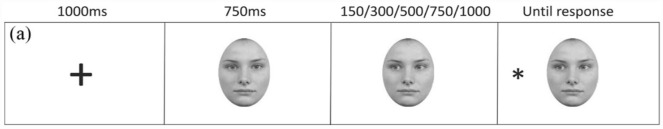
Overview of the cueing paradigm in Experiment 1 of Gregory and Jackson (2020). *Source.* This figure has been adapted from Gregory and Jackson (2020).

We extracted trials from Experiment 1 of Gregory and Jackson (2020), where a total of 41 (6 males, 35 females) participants were recruited (see Figure 2 for detail on the procedure and stimuli). There were three types of cues used in the original study, with 240 trials per cue type: gaze cues, arrow cues, and line cues (where the cue was an off-centre vertical line placed over a longer horizontal line) but for the current study we only analysed gaze cues.^4^ Other manipulations in the original study were within-subject cue validity (cued or miscued) and SOA (100, 300, 500, 700, and 1,000 ms). We ignored the distinction between SOA in our analyses to keep consistency across data sets. It is worth pointing out recent meta analytic findings which suggest that the magnitude of the gaze cueing effect is equivalent across these SOAs, suggesting that there may not be any moderating effect (McKay et al., 2021). However, similar magnitudes does not necessarily mean there are no differences in underlying mechanisms (although see Supplementary Material 11 where we do model the gaze cueing effect at each level of SOA in another data set and find no qualitative evidence for moderation).

No participants were excluded, as they all had an accuracy of >80% (*M* = 98%). However, 14 trials (<.01%) were removed for response times being <100 ms or >5,000 ms, leaving a total of 9,826 trials analysed in Data Set 1.

#### Data Set 2: Carlson (2016)

**Figure 3.**
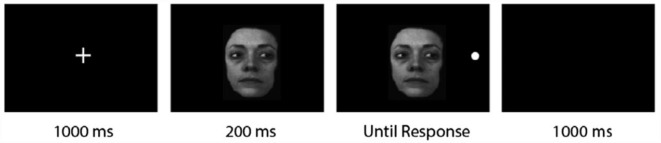
Overview of the cueing paradigm in Carlson (2016). *Source.* This figure has been adapted from Carlson (2016).

Fifty-one participants were recruited, however, we only received data for the 50 participants who were included in the original study (25 males, 25 females), with the original author excluding one participant whose response time was more than three standard deviations from the group mean. There were three cue validity conditions: cued, miscued, and forward (where the eye gaze was forward rather than left or right). In the current study, we did not analyse forward trials, only cued or miscued trials because the forward gaze cues were non-directional. Carlson (2016) also manipulated facial expression of the cue (fearful or neutral). However, we ignored the distinction between facial expressions in analyses to facilitate consistency across data sets. Figure 3 provides more detail on their procedure and stimuli. No participants were removed as they all had an accuracy of >80% (*M* = 99%), but 14 trials (<.01%) were removed for response times being <100 ms or >5,000 ms, leaving 12,786 trials subject to analysis.

#### Data Set 3: Chen et al. (2021)

**Figure 4.**
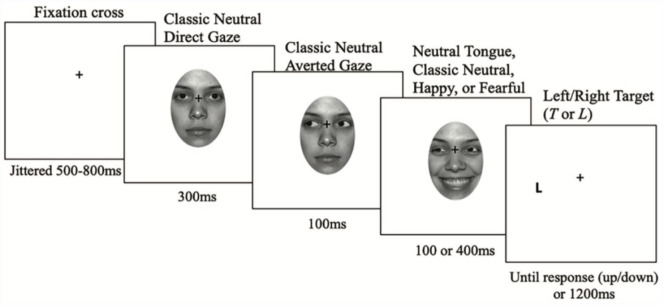
Overview of the cueing paradigm in Chen et al. (2021). *Source.* This figure has been adapted from Chen et al. (2021).

A total of 80 participants were recruited (Figure 4 provides more detail on their procedure and stimuli). Eight participants were removed: three who were marked as having errors during data collection by the original authors and five who had an accuracy of less than 80% (*M* = 94%). One participant was missing from the raw data, leaving data for 71 participants remaining.^5^ From the remaining participants, 1,974 trials (2%) were removed for response times being <100 or >5,000 ms, and one participant only completed two-thirds of the task (but their responses were still analysed), leaving a total of 106,570 trials subject to analyses.

Although we only used data from the localisation task, it is worth noting that participants also completed a discrimination task in separate blocks, whereby they had to determine the letter that the target was. Furthermore, in addition to a cue validity manipulation (cued vs. miscued trials), Chen et al. also had within-subject cue facial expression (fearful, classic neutral, neutral tongue, happy) and cue–target SOA (100, 400 ms) manipulations; however, for consistency across data sets, we ignored the distinctions between those trials. However, in Supplementary Material 7, we report individual-level model comparisons at each level of SOA and cue facial expression and do not find any clear moderating effects.

### Implementation of analyses

We analysed the data in three stages (described in detail in the following sections). In Stage 1, we performed a simple descriptive analysis in order to identify which participants exhibited cueing magnitudes in a direction consistent with a positive cueing effect (i.e., faster responses for cued trials compared with miscued trials). In Stage 2, we fitted eight variants of the DDM to each participant’s data. Different model variants allowed different subsets of parameters to vary across cuing conditions. By comparing the performance of each model using model selection criteria, we were able to identify which model performed best and by extension, assess the relative importance of allowing each parameter to vary with cueing. Importantly, Stage 2 provided an opportunity to examine any individual differences in the processes driving the emergence of any positive gaze cueing effects. In Stage 3, we identified whether parameter estimates varied at the group-level across cued and miscued trials using a hierarchical model where the parameters each had equal opportunity to vary across cuing conditions. This group-level analysis provided another method of identifying subserving cognitive mechanisms because shifts in group-level parameter estimates that correspond to systematic differences in response times across conditions may suggest that those parameter shifts are driving observed differences in response times. Furthermore, this method allowed us to asses the uncertainty of any shifts in parameter estimates at the group level.

#### Stage 1: cueing magnitudes

All of the pre-existing data sets used in the current study published significant (*p* < .05), group-level cueing effects, such that their participants had faster response times for cued compared with miscued trials. None of these studies investigated individual differences in the emergence of cueing effects within participants. As described above, we identified mean response time differences for each participant (hereafter, “cueing magnitudes”). Identifying individuals who showed cueing effects in this way was important to provide context for the individual-level computational modelling analyses described later since we did not expect the same models to perform best for participants who responded slower to cued trials compared with miscued trials. Separating gaze cueing magnitudes by subject also provided useful insight on its own because the gaze cueing effect is rarely investigated at the individual level.

In addition to calculating gaze cueing magnitudes for each individual, we also calculated the mean and *SD* of the gaze cueing magnitudes associated with each data set (i.e., at the sample level). Furthermore, to facilitate comparison between data sets, we calculated the standardised mean change score (Gibbons et al., 1993) for each Data Set using the “metafor” package in R (Viechtbauer, 2021).

#### Stage 2: individual level modelling

In this stage, we investigated the relative importance of each of the parameters of interest (non-decision time, starting point, and drift rate) by comparing predictions generated by eight variants of the DDM (Ratcliff & McKoon, 2007) against each individual participant’s data.^6^ Each of the eight models varied based on which parameters were permitted to vary across cued and miscued trials. Importantly, although certain parameters were allowed to vary across cued and miscued trials, they were constrained by what was theoretically plausible. Specifically, the constraints were based on the assumption that there would be a positive gaze cueing effect (i.e., faster response times for cued compared with miscued trials). We provide an overview of each model and their constraints in the following section. In addition, Table 2 describes precisely the parameter constraints within each model.

**Table 2.** Parameter constraints for each individual-level model.

Model	Parameters			
	Non-decision time (t0)	Starting point (z)	Drift rate (v)	Threshold (a)
Simple	$t0_{c}=t0_{m}$	0.5	$v_{c}=v_{m}$	$a_{c}=a_{m}$
t0	$t0_{c}<t0_{m}$	0.5	$v_{c}=v_{m}$	$a_{c}=a_{m}$
z	$t0_{c}=t0_{m}$	$z_{c}>0.5,z_{m}=1-z_{c}$	$v_{c}=v_{m}$	$a_{c}=a_{m}$
v	$t0_{c}=t0_{m}$	0.5	$v_{c}>v_{m}$	$a_{c}=a_{m}$
z-v	$t0_{c}=t0_{m}$	$z_{c}>0.5,z_{m}=1-z_{c}$	$v_{c}>v_{m}$	$a_{c}=a_{m}$
t0-v	$t0_{c}<t0_{m}$	0.5	$v_{c}>v_{m}$	$a_{c}=a_{m}$
t0-z	$t0_{c}<t0_{m}$	$z_{c}>0.5,z_{m}=1-z_{c}$	$v_{c}=v_{m}$	$a_{c}=a_{m}$
Complex	$t0_{c}<t0_{m}$	$z_{c}>0.5,z_{m}=1-z_{c}$	$v_{c}>v_{m}$	$a_{c}=a_{m}$

$c^{=}$ cued trials, $m^{=}$ miscued trials

##### Simple model

This was the standard DDM. None of the parameters were permitted to vary across cued and miscued trials. Effectively then, this model represented the “null model” as it assumed there were no meaningful differences in response time across cued and miscued trials due to the mechanisms proposed in the models, or no meaningful differences in response times in the behavioural data.

##### t0 model

Within the t0 model, non-decision time was estimated separately for cued and miscued trials, but was constrained such that non-decision time could not be longer for cued trials than it was for miscued trials. That is, this model implemented an orienting benefit for targets appearing at the cued location. No other parameters varied across conditions.

##### z model

Within the z model, starting point was estimated separately for cued and miscued trials but was constrained such that starting point could not be shifted to be closer to the gazed away from location than the gazed at location. That is, this model implemented a decision bias favouring responses indicating the cued location. No other parameters varied across conditions.

##### v model

Within the v model, drift rate was estimated separately for cued and miscued trials but was constrained such that drift rate could not be lower for cued trials than for miscued trials. This model implemented more efficient and higher-quality processing for targets appearing at the cued location.

##### z-v model

The z-v model permitted both z and v to vary independently across cued and miscued trials with the same constraints as the single parameter models.

##### t0-z model

As for the z-v model, but it was t0 and z that were allowed to vary across cued and miscued trials rather than z and v.

##### t0-v model

As for the z-v model, but it was t0 and v that were permitted to vary across cued and miscued trials rather than z and v.

##### Complex model

Within the complex model, t0, z, and v were permitted to vary independently across cued and miscued trials with the same constraints as the single parameter models.

We used a Bayesian parameter estimation method—specifically, differential evolution Markov chain Monte Carlo (Braak, 2006) 3k with chains (where k is the number of free parameters in each model) and 4,000 iterations, with the first 2,000 iterations discarded as burn-in—to calculate the maximum likelihood estimates of each of the eight model variants specified above. The R package “rtdist” (Singmann et al., 2020) was used for the likelihood function of the diffusion model, and the R package “msm” (Jackson, 2021) was used to calculate the likelihood function of the truncated normal distribution. We chose this Bayesian approach because Bayesian methods can provide a better ability to traverse complex likelihood spaces and avoid local maxima through moving both up and down the likelihood surface (as found in Evans, Dutilh, et al., 2020).

We used reasonably uninformed priors to ensure that the maximum likelihood would be contained within the posterior (see exact priors in Supplementary Material 1). We took the maximum likelihood as the parameter set with the highest probability of the data given the parameters of the estimated posterior distribution.

##### Inference methods

In this stage we used two main inference methods, which we describe briefly here and in detail over the following sections. The first method, weighted model probabilities, allowed us to get a big picture understanding of what the preferred model was by directly evaluating the performance of each model relative to each of the other models (or other *candidate* models). Comparatively, the second method, parameter inclusion probabilities, provided a more detailed perspective of how important each of the three parameters of interest were for describing the observed data.

##### Weighted model probabilities

The purpose of this inference method was to identify the probability of each model being the best model relative to the other candidate models. We fitted the models to each participant separately and evaluated the models’ performances using two respective model selection criteria: Bayesian information criterion (BIC) and Akaike information criterion (AIC). BIC and AIC are both probabilistic model comparison methods that make different trade-offs between the quantitative ability of a model to describe the data (or model “fit”) and model complexity (or “parsimony”), with one key difference between criteria being that BIC penalises complexity more than AIC (for more information on the differences between each criterion see Evans, 2019). We then converted the model selection criteria scores into weighted probabilities (see Wagenmakers & Farrell, 2004) as this allowed us to better assess the uncertainty in each criterion’s scores and the relative performance of each model.

##### Parameter inclusion probabilities

After assessing the weighted probabilities of each respective model, we then assessed the relative influence of each parameter of interest (non-decision time, starting point, and drift rate). This was achieved by combining the relative probability, according to BIC, of models that contained the same assumption about each respective parameter (see Boehm et al., 2021, note that only the BIC parameter inclusion probabilities are presented in this manuscript as the qualitative trends were equivalent for AIC, but the AIC inclusion probabilities can be found in Supplementary Material 6). We then compared them with models that do not make the same assumption in order to identify the relative importance of that assumption. For example, to assess the inclusion probability for the parameter v, we compared the relative performance of models that assume v varies across cue validity conditions (v, v-t0, z-v, and complex) with models that did not assume a drift rate difference across validity conditions (z, t0, z-t0, and simple).

#### Stage 3: Bayesian hierarchical modelling

In this stage, rather than comparing the performance of competing models, we identified differences in parameter estimates across cued and miscued trials for just one model—the complex DDM variant described above, which allowed each of the parameters to vary across cued and miscued conditions. If a parameter is explaining differences in response times across cued and miscued cue trials, then there should be reliable differences in the estimates of those parameters across those conditions. Similarly, if a parameter is not explaining any differences in response time across cued and miscued trials, then there should be no reliable differences between estimates across those conditions (for more detail on estimation approaches vs. model selection approaches to inference, see Kruschke, 2011; Kruschke & Liddell, 2018).

To make inferences about the magnitude of these parameter differences, we compared the distributions of the differences in parameter estimates across cued and miscued trials. We also assessed the mean differences and the 95% Bayesian credible intervals, which we use to assess the variability in the distribution of posterior parameter estimates and gauge the probability that there were meaningful (or credible) differences in parameter estimates across conditions. If zero lies within the 95% credible interval, there is less than 95% probability that there were reliable differences across parameter estimates in cued and miscued trials. Importantly, we used hierarchical Bayesian parameter estimation. In hierarchical Bayesian parameter estimation, each participant’s parameters are constrained to follow a group-level distribution. Here, the group-level pertains to the trial type (i.e., miscued trial or cued trial). Hierarchical parameter estimation is advantageous because individual participants mutually inform parameter estimates of one another.

The constraints on this model were different from the constraints that were placed on the individual-level complex model. In the individual-level modelling stage, we fully constrained the models so that they could not make theoretically implausible predictions. However, that would not work for these analyses where we were making inferences about parameter shifts across conditions (rather than directly comparing the performance of models), because if the models were completely constrained, the parameter estimates would be forced to shift across conditions even if they were not driving the effect. In other words, constraining the parameter estimates effectively prevents the models from identifying no differences because virtually all of the probability mass is forced to take on non-zero values. Because hierarchical methods pool parameter estimates on both the group level and the individual level, parameters can be constrained on either of those levels. However, if we forced each individual’s prediction to only vary in the theoretically plausible direction, it would ensure that no single individual’s differences in parameter estimates could cross zero, making it much less likely that the group-level parameter estimates would cross zero, even if there were no differences in that parameter across conditions. Therefore, for this analysis, the group-level parameter estimates were unconstrained. Importantly though, we ran a unique model for each parameter of interest in each data set, where on the individual level, all of the parameters, except for the specific parameter of interest for that model, was constrained.^7^ For example, for the parameter z in Data Set 1, we ran a model where the individual-level estimates of t0 and v were constrained, but the estimates for z were completely unconstrained (this process was then repeated for to and v for each dataset). We then looked at the parameter estimates for the unconstrained parameter to see whether it reliably shifted in a theoretically plausible direction. This process meant that the models were constrained enough to not make completely theoretically implausible predictions, but were still flexible enough such that the parameters could actually cross zero if they were not driving the effect.

As with the individual-level estimates, we used reasonably uninformed priors (see exact priors in Supplementary Material 3). The R package “rtdist” (Singmann et al., 2020) was used for the likelihood function of the diffusion model, and the R package “msm” (Jackson, 2021) was used to calculate the likelihood function of the truncated normal distribution.

## Results and discussion

### Data Set 1

#### Stage 1: cueing magnitudes

**Figure 5.**
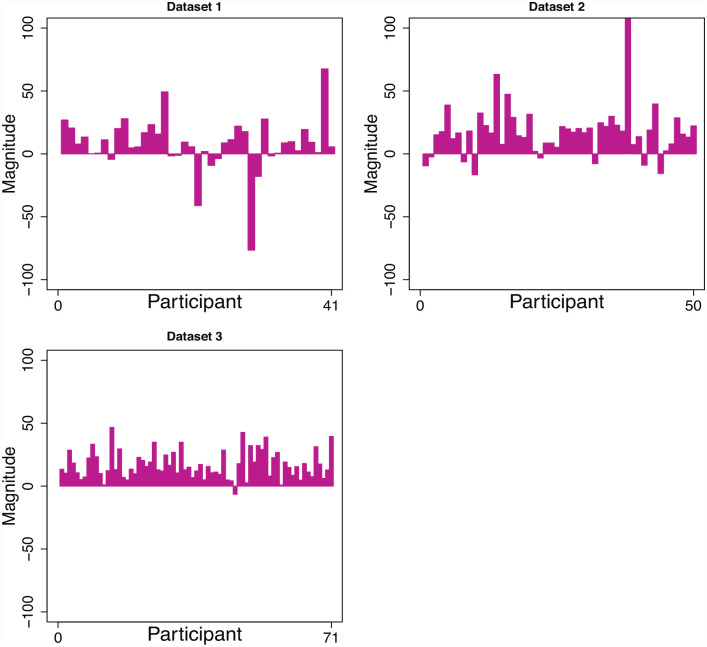
Cueing magnitudes in milliseconds for each participant and data set. Positive cueing magnitudes indicate faster mean responses to the cued location.

In all 32 out of the 41 participants had faster mean response times for cued trials compared with miscued trials (see Figure 5). The mean cueing magnitude for Data Set 1 including all 41 participants was 8 ms (*SD* = 2 ms; standardised mean change = 0.18).

#### Stage 2: individual-level modelling

A visualisation of the model predictions plotted against participant data from Data Set 1 (i.e., the model fits) is given in Supplementary Material 5. Assessment of these fits revealed all of the models seemed to provide a reasonably good account for the data, except for the simple model, which could not account for the qualitative group-level differences in response times across across cued and miscued trials, and the v model, which under-estimated earlier response time differences across cued and miscued trials. Models that permitted t0 to vary across conditions appeared to be the only models that could account for a shrinkage in the effect later in the response time distribution, although there was still some misfit in those predictions. Specifically, they tended to over-predict the variance of the response time distributions such that they predicted response times earlier in the distribution should be faster than what was observed and response times later in the distribution should be slower than what was observed.

##### Weighted model probabilities

**Figure 6.**
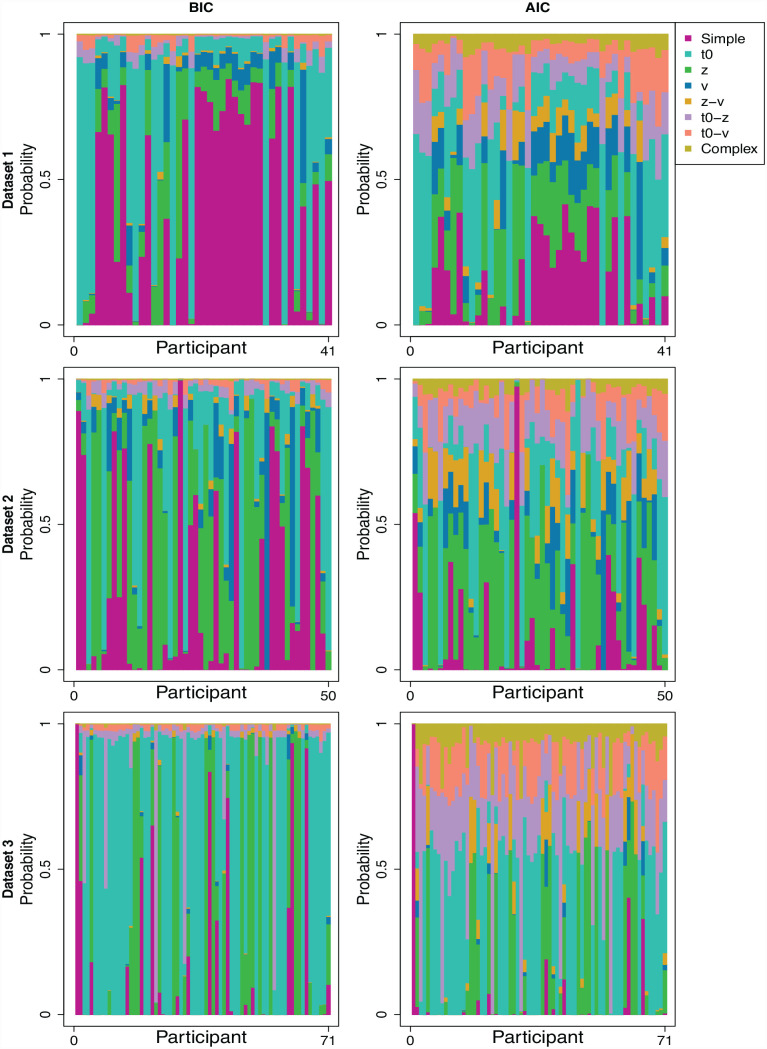
BIC and AIC weighted model probabilities for each participant and data set. Collapsing across all data sets and participants, according to BIC, the t0 model had the highest relative probability at 40%, followed by the Simple model at 24%, and the z model at 23% (all other models had lower than 5% probability). According to AIC, the t0 model had the highest probability at 28%, followed by the z model at 19%, and the z-t0 model at 18% (all of the other models had a probability of 10% or lower).

The top panel in Figure 6 shows a graphical representation of the probability that each model is the best performing model for each participant in Data Set 1 according to BIC and AIC. Table 3 shows the raw BIC and AIC values collapsed across participants as well as the exact weighted BIC and AIC probabilities collapsed across participants.

**Table 3.** Model performance as indicated by the individual-level modelling for Data Set 1 collapsed across participants.

Model	BIC		AIC	
	Probability (%)	Raw score	Probability (%)	Raw score
Simple	42	−505.45	15	−515.92
t0	35	−508.06	29	−522.02
z	11	−505.19	15	−519.15
v	6	−502.28	9	−516.25
z-v	1	−499.66	6	−517.12
t0-v	2	−502.83	11	−520.28
t0-z	2	−503.06	11	−520.52
Complex	0	−497.57	4	−518.52

BIC: Bayesian Information Criterion; AIC: Akaike information criterion.

“Probability” represents the relative probability of each model being the best candidate model according to BIC and AIC, respectively. “Raw Score” corresponds to the raw BIC and AIC scores.

Higher probabilities indicate a higher probability of that model being the best performing model. Lower raw BIC and AIC scores indicate better model performance. To get the values shown in this table, we calculated a unique raw AIC and BIC and weighted AIC and BIC for each participant and model, and then calculated the average values by collapsing these individual scores across participants.

According to BIC as shown in Figure 6, 22 participants were best described by the simple model, 15 participants were best described by the t0 model, 3 by the z model, and 1 by the v model. When collapsing across participants and taking the mean, as shown in Table 3, according to BIC the Simple model had the highest probability of being the best performing model, followed by the t0 model, although t0 still had the best raw BIC score.

According to AIC, 18 participants were best described by the t0 model, 12 by the simple model, 9 by the z model, and 2 by the v model. When collapsing across participants, as shown in Table 3, according to AIC, the t0 model was most likely to be the best performing candidate model followed by the simple model.

The key difference between model selection criteria was that BIC was favouring the simple model substantially more than AIC. This makes sense given BIC penalises model flexibility more than AIC does (Evans, 2019) and the simple model is nested within the t0 model, so the t0 model is inherently more flexible. Visual inspection of Figure 6 confirms that the qualitative predictions of each selection criterion was comparable, whereby the same participants who had a high probability of t0 being their best performing model according to BIC also had a high probability according to AIC.

The good performance of the simple model is likely driven by the large proportion of cueing magnitudes that were close to zero or negative in Data Set 1 (see Figure 5). Supporting this explanation, there was a negative correlation between the probability of participants being best described by the simple model and their cueing magnitudes (*r* = −.55, *BF* = 163.62), such that participants who had a higher probability of being best described by the simple model tended to have to have smaller cueing magnitudes. The model fits in Supplementary Material 5 further corroborate this assessment; although the simple model performed well according to BIC, it could not account for the group-level qualitative differences in response times across cued and miscued trials. This suggests that when considering which model best described any *cueing effects* in the data, the t0 model was likely the best model.

Given these small to negative cueing magnitudes, we suggest that much of the individual variability shown in this data is most likely due to noise, so we will not make any strong interpretations about individual differences in the processes driving this data; however, given the presence of individual differences, future studies should explore the possibility.

##### Parameter inclusion probabilities

**Figure 7.**
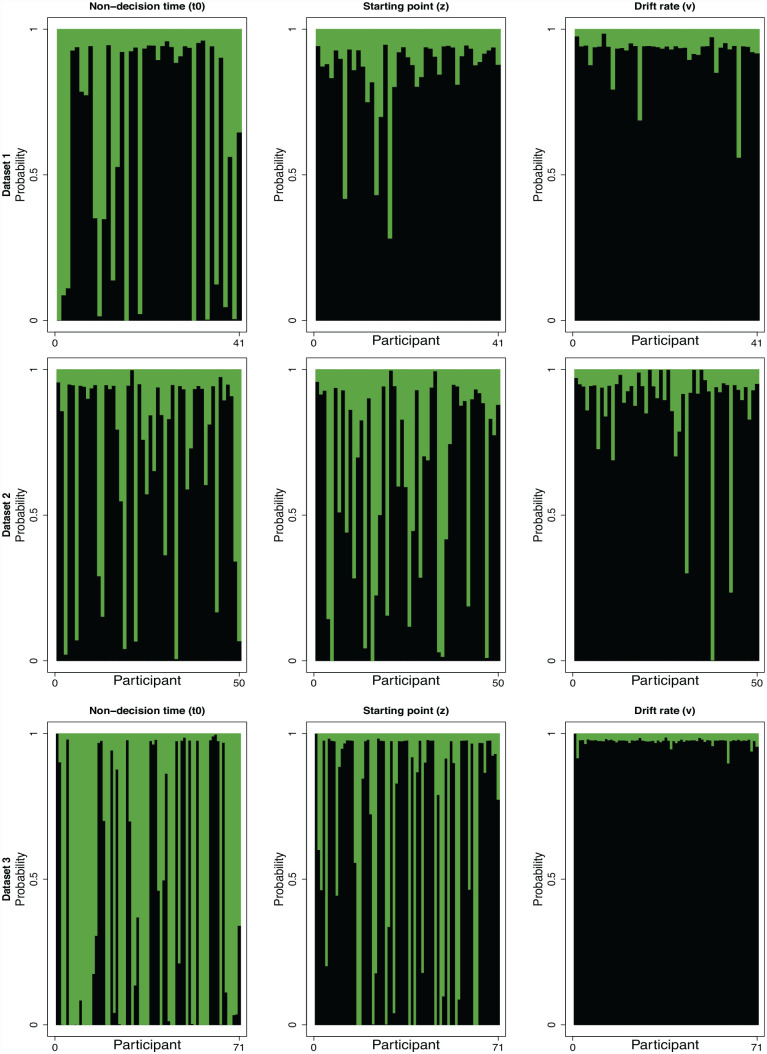
BIC parameter inclusion probabilities for each participant and data set. Collapsing across data sets and participants, models that allowed t0 to vary across conditions were most probably at 40%, followed by z at 30% and v at 7%. Green represents models that assume the respective parameter varies across conditions. Larger proportion of green in these figures indicates broader support for a parameter driving differences across cued and miscued conditions.

As shown in the top panel of Figure 7, 15 participants had a greater than 50% chance of being best described by models that permitted t0 to vary across conditions. Three participants had a greater than 50% chance of being best described by models that permitted z to vary across conditions. One participant had a greater than 50% chance of being best described by models that permitted v to vary across conditions.

Collapsing across participants, models that assumed t0 varied across cued and miscued trials were on average 40% likely to be the best candidate model when compared with models that did not make this assumption (z, v, z-v, simple; see Figure 7, top panel). Models that assumed z varied across cued and miscued trials were on average 14% likely to be the best candidate model compared with models that did not make this assumption. Models that assumed v varied across cued and miscued trials were on average 9% likely to be the best candidate model compared with models that did not make this assumption.

Although on average, no single parameter assumption had more than a 50% probability of describing the data, this is because the simple model performed so well (which is, as we discussed in the “Weighted Model Probabilities” section above, most likely due to the cueing magnitudes being very small). However, when comparing the parameters of interest against each other by comparing the green in Figure 7, models that included the assumption that t0 varies across conditions tended to perform better compared with models that included the assumption that z or v varied. Therefore, these analyses suggest t0 was the most likely out of the parameters of interest to be driving the positive cueing effects in the observed data.

#### Stage 3: Bayesian hierarchical modelling

**Figure 8.**
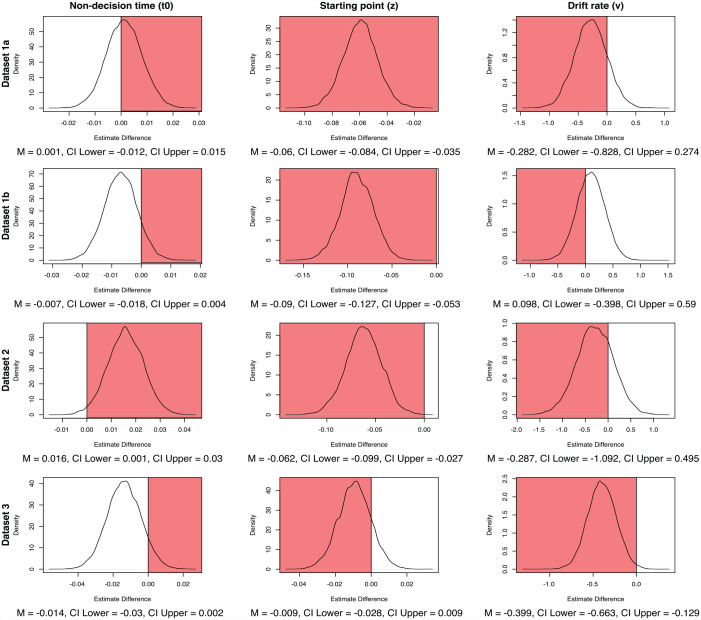
Differences between hierarchical Bayesian parameter estimate distributions across cued and miscued trials.

As shown in the top panel of Figure 8, none of the mean differences in parameter estimates showed a 95% credible difference from zero in the theoretically plausible direction. Indeed, on average all parameters shifted in the opposite direction to what was theoretically plausible. Although t0 was the closest to credibly shifting in the theorised direction, the estimates were still firmly centred around zero. These null estimates likely reflect the large amount of small and negative cueing effects in Data Set 1 (see Figure 5) and are largely consistent with what we found in the individual-level modelling, whereby there was evidence that the simple model performed best when collapsing across participants.

The red portion of the plots represent the space of parameter differences which is inconsistent with the theoretically plausible direction for each parameter assuming a positive cueing effect, as outlined in the Table 2. Specifically, in order to be considered theoretically plausible, t0 needed to show a negative difference across cued and miscued trials, whereas v and z needed to show positive differences. This plot shows a large proportion of differences close to zero and in the theoretically implausible direction, which we attribute to variability on the individual level.

### Data Set 2

#### Stage 1: cueing magnitudes

In all, 42 out of the 50 participants displayed faster response times for cued compared with miscued trials, as shown in Figure 4. The mean cueing magnitude for Data Set 2 including all 50 participants was 18 ms (*SD* = 25; standardised mean change = 0.26).

#### Stage 2: individual-level modelling

A visual depiction of the model predictions plotted against participant data from Data Set 2 can be found in Supplementary Material 5. Assessment of these predictions revealed similar qualitative trends to those in Data Set 1, but with greater misfit in the earlier and later quantiles such that the models tended to over-predict the variance in the response time distributions. As before, the models overpredicted variance in response times across the response time distribution. Specifically here, the models predicted faster response times compared with what was observed earlier in the response time distribution and slower response times compared with what was observed later in the distribution.

##### Weighted model probabilities

According to BIC and as shown in Figure 6, 18 participants were best described by the z model, 17 by the simple model, 12 by the t0 model, and 3 by the v model. As shown in Table 5, averaged across participants, the z model was the most likely to be the best performing candidate model followed by the simple model. According to AIC, 20 participants were best described by the z model, 15 by the t0 model, 4 by the v model, and 1 by the t0-v model. Averaging across participants, the z model was the most likely to be the best performing candidate model followed by the t0 model (Table 4).

**Table 4.** Model performance as indicated by the individual-level modelling for Data Set 2 collapsed across participants.

Model	BIC		AIC	
	Probability (%)	Raw score	Probability (%)	Raw score
Simple	27	−608.93	10	−619.56
t0	24	−612.10	21	−626.27
z	33	−615.07	29	−629.25
v	9	−609.64	9	−623.82
z-v	2	−607.38	8	−625.10
t0-v	2	−606.77	8	−624.49
t0-z	3	−610.32	12	−628.04
Complex	0	−602.46	4	−623.73

BIC: Bayesian information criterion; AIC: Akaike information criterion.“Probability” represents the relative probability of each model being the best candidate model according to BIC and AIC, respectively. “Raw score” corresponds to the raw BIC and AIC scores.

Higher probabilities indicate a higher probability of that model being the best performing model. Lower raw BIC and AIC scores indicate better model performance.

##### Parameter inclusion probabilities

As shown in the third panel of Figure 7, 11 participants had greater than 50% chance of being best described by models that permitted t0 to vary across conditions. Sixteen participants had greater than 50% chance of being best described by models that permitted z to vary across conditions. Three participants had greater than 50% chance of being best described by models that permitted v to vary across conditions.

Collapsing across participants, models that assumed t0 varied across cued and miscued trials were on average 28% likely to be the best candidate model when compared with models that did not make this assumption. Models that assumed z would vary across cued and miscued trials were on average 38% likely to be the best candidate model compared with models that did not make this assumption. Models that assumed v varied across cued and miscued trials were 13% likely to be the best candidate model compared with models that did not make this assumption.

The results of these individual-level analyses suggest z was the most likely out of the parameters of interest to be explaining any positive cueing effects in the data. These results generally conflict with what was found in Data Set 1. In Data Set 1, t0 tended to have the highest probability of underlying positive cueing effects for most participants, although a small minority of participants were also best described by z. Notably though, in Data Set 2 there was still a large proportion of participants whose data was best explained by the t0 model.

#### Stage 3: Bayesian hierarchical modelling

As shown in the second panel of Figure 8, none of the parameters showed reliable differences in the theoretically plausible direction. The mean estimate for v was the most likely to be credibly different from zero in the theoretically plausible direction, but the distribution of estimates was still firmly centred around zero. As discussed in relation to Data Set 1, we suggest that these lack of differences in this group-level modelling is likely due to the high level of individual variability in Data Set 2, with most participants being best described by z, but still a high proportion of participants also being best described by t0.

### Data Set 3

#### Stage 1: cueing magnitudes

In all, 70 out of the 71 participants displayed faster response times for cued trials compared with miscued trials, as shown in Figure 4. The mean cueing magnitude for Data Set 3 including all 71 participants was 17 ms (*SD* = 11 ms; standardised mean change = 0.63).

#### Stage 2: individual-level modelling

A visual depiction of the model predictions plotted against participant data from Data Set 3 can be found in Supplementary Material 5. As with the other datasets, all of the models seemed to provide a reasonably good account for the data except for the simple model which could not account for the qualitative differences in response times across across cued and miscued trials, and the v model, which under-estimated differences across cued and miscued trials at earlier response times.

##### Weighted model probabilities

According to BIC, as shown in Figure 6, 42 participants were best described by the t0 model, 17 by the z model, 7 by the simple model, and 5 by the t0-z model. As shown in Table 5, averaged across participants, the t0 model was the most likely to be the best performing model followed by the z model. According to AIC, as shown in Figure 5, 39 participants were best described by the t0 model, 19 by the z model, 10 by the z-t0 model, 2 by the simple model, and 1 by the t0-v model. As shown in Table 5, averaged across participants, the t0 model had the highest probability of being the best performing model followed by the t0-z model.

**Table 5.** Model performance as indicated by the individual-level modelling for Data Set 3 collapsed across participants.

Model	BIC		AIC	
	Probability (%)	Raw score	Probability (%)	Raw score
Simple	9	−1,482.12	2	−1,498.06
t0	56	−1,521.57	33	−1,542.82
z	24	−1,505.63	16	−1,526.88
v	1	−1,481.10	1	−1,502.35
z-v	1	−1,498.04	5	−1,524.60
t0-v	1	−1,514.79	11	−1,541.36
t0-z	8	−1,521.25	25	−1,547.82
Complex	0	−1,513.46	7	−1,545.34

BIC: Bayesian information criterion; AIC: Akaike information criterion.“Probability” represents the relative probability of each model being the best candidate model according to BIC and AIC, respectively. “Raw score” corresponds to the raw BIC and AIC scores.

Higher probabilities indicate a higher probability of that model being the best performing model. Lower raw BIC and AIC scores indicate better model performance.

##### Parameter inclusion probabilities

As shown in the third panel of Figure 7, 41 participants had a greater than 50% chance of being best described by models that permitted t0 to vary across conditions; 16 participants had a greater than 50% chance of being best described by models that permitted z to vary across conditions. No participants had a greater than 50% chance of being best described by models that permitted v to vary across conditions.

Collapsing across participants, models that assumed t0 varied across cued and miscued trials were on average 66% likely to be the best candidate model when compared with models that did not make this assumption. Models assuming that z would vary across cued and miscued trials were on average 33% likely to be the best candidate model compared with models that did not make this assumption. Models that assumed v varied across cues and miscued trials were 3% likely to be the best candidate model compared with models that did not make this assumption.

Overall, these individual-level results are consistent with what was found in Data Set 1. Again, t0 was most likely to be underlying cueing effects when collapsing across participants, but we found further evidence for individual variability, whereby a reasonable subset of participants were best described by z and a smaller subset were best described by both t0 and z. Although we did find similar individual variability in the other data sets, those found in this data set were by far the most credible, as shown by the high probability that either z or t0 provided the best explanation for participants’ data (see Figures 6 and 7; particularly, note the high proportion of green and light blue for participants in Data Set 3, Figure 6 and the high proportion of green compared with black for participants in Data Set 3, Figure 7 relative to the other data sets). These findings make sense since Data Set 3 was the most suited for examining individual differences given that it had by far the most trials per participant (Data Set 3 had more trials than all of the three other data sets combined) as well as the most participants. As such, given these results, we are at this stage able to conclude that there appear to be individual differences in the mechanisms underlying people’s responses to gaze cues whereby for most people, t0 provides the best explanation for their data, whereas others are best described by z, and an even smaller subset by both t0 and z.

#### Stage 3: Bayesian hierarchical modelling

As shown in the second panel of Figure 8, t0 was the only parameter to show a mean difference in estimates in the theoretically appropriate direction across cued and miscued trials. Although this difference was not 95% credibly different from zero, the vast majority of the distribution indicated a shift in the theoretically plausible direction. The mean difference in estimates for z was the next closest to credibility followed by v, but both of their estimates were firmly in the theoretically implausible direction.

Largely consistent with many of the results found so far, these results suggest that t0 was the parameter most likely to be driving any differences between cued and miscued trials, but there was a reasonable degree of uncertainty. As discussed in relation to the other data sets, we suggest that this uncertainty is likely due to variability at the individual level. Notably though, as can be seen by the relatively low proportion of the distribution overlapping zero in the bottom left plot in Figure 8, there was less uncertainty in the hierarchical group-level estimates of Data Set 3 compared with the other data sets, which makes sense considering Data Set 3 had the least individual variability and the highest probability of participants being best described by t0 on the individual level.

## General discussion

The gaze cueing effect has been widely studied and is considered as a critical component of social cognition (e.g., see Frischen et al., 2007; Stephenson et al., 2021). Despite this, relatively little research has sought to understand the cognitive processes underlying its emergence. Evidence accumulation models represent the dominant framework through which the cognitive processes underlying speeded decisions can be inferred (Donkin & Brown, 2018; Evans & Wagenmakers, 2019; Forstmann et al., 2016; Ratcliff et al., 2016) and have recently been identified as an important (but underused) tool for studying social cognition (Parker & Ramsey, 2022b). Here, we focused on three particular cognitive mechanisms described by evidence accumulation models: non-decision time, starting point, and drift rate. We applied theoretically informed variants of the DDM evidence accumulation model to three pre-existing gaze cueing data sets with the aim of better understanding the cognitive mechanisms underlying the gaze cueing effect.

Overall, at the group level and when collapsing across individual participants, we found that the gaze cueing effect was generally best described by an attentional orienting account (the non-decision time parameter in the DDM) rather than a transient information processing account (the starting point parameter in the DDM) or sustained information processing account (the drift rate parameter in the DDM). Specifically, our findings suggest that for most people, gaze cues tended to elicit an early, reflexive shift of attention to the gazed at location which resulted in a response time benefit when the target appeared at the gazed-at location and a response time orienting cost when the target appeared at the gazed-away from location. This is consistent with previous characterisations of the gaze cueing effect as a covert, reflexive shift of attention to the gazed at location (e.g., Friesen & Kingstone, 1998; Frischen et al., 2007; McKay et al., 2021; McKay et al., 2022; Sato et al., 2007) as well as orienting theories of attention that describe orienting and processing as a serial process, whereby people must first orient towards the target before they can process it.

Although we found that non-decision time provided the best account for the gaze cueing effect overall, we also found considerable individual variability, such that some people’s gaze cueing effects were best described by the starting point parameter. This was particularly evident in Data Sets 2 and 3, where almost everyone showed a positive gaze cueing effect, but what tended to vary across participants was whether the gaze cueing effects were better described by non-decision time or starting point. These data sets also had the most trials per person, the most participants, and the largest gaze cueing effects, suggesting that this individual variability might represent credible individual differences. Previous research has suggested that gaze cueing effects could be representative of both an initial reflexive attentional orienting response followed by a top down, decision-level response (Hill et al., 2010), which one could interpret as requiring increased allocation of information processing resources to cued locations. Our findings generally do not support this explanation, however, as most people tended to be best described by either an attentional-orienting account or a resource allocation account, but models that integrated both of these perspectives very rarely performed well.

For those participants whose gaze cueing effects were best described by starting point, there are two ways that this could have occurred according to evidence accumulation models. One common interpretation of starting point shifts is that people had an a priori expectation that the target would appear at the gazed-at location before it actually appeared (Voss et al., 2004; White & Poldrack, 2014). This would suggest that, consistent with the view that eye-gaze is a highly informative social cue people innately use to infer the intentions of others (Calder et al., 2002; Emery, 2000; Pellicano & Rhodes, 2003), these participants believed that the cues were informative, so they adjusted their expectation to account for where the target was likely to appear. However, given that the gaze cueing effects analysed in this study were non-predictive (there was an even amount of cued and miscued trials), this interpretation seems unlikely, since there was no reason for participants to believe that the cues were informative of the actual location of the target.

The interpretation that we think is most likely given the tasks used in the current study is that shifts in starting point were indicative of short-lived allocation of information processing resources to the cued location. This interpretation of starting point comes from two-stage evidence accumulation theories that describe the first stage of evidence accumulation as an initial, quick burst of information processing—which in the standard DDM used in our study would be captured by starting point—followed by a second stage of slower, sustained evidence accumulation which is captured by drift rate (Weigard et al., 2019), and is consistent with resource allocation theories of visual attention that suggest attention acts as a mechanism for distributing limited cognitive resources (see Sewell & Smith, 2012). This finding is also broadly consistent with research in joint attention suggesting people allocate more information processing resources to gazed-at locations (Reid et al., 2004; Reid & Striano, 2005; Shteynberg, 2015; Wahl et al., 2013, 2019). Our findings suggest that for some people (i.e., those best described by non-decision time), gaze cueing effects are driven purely by orienting costs, where a time cost occurs because people need to reorient to the cued location *before* they begin to process the target (Broadbent, 1958; Sewell & Smith, 2012). Other people, however (i.e., those best characterised by starting point), seemed to engage in attentional orienting and information processing in parallel, whereby the gaze cue prompts them to simultaneously orient attention and allocate information processing resources to the cued location (Sperling & Weichselgartner, 1995). After the initial presentation of the cue, however, people best characterised by starting point then gradually redistribute their information processing resources more evenly across the visual field.

It remains unclear precisely why these individual differences in the mechanisms underlying gaze cueing effects have emerged, as there are very few previous studies that have looked at individual differences in the gaze cueing effect in healthy adult populations. Some studies have found that self-reported sex-category membership modulates the magnitude of gaze cueing effects. Specifically, participants identifying themselves as female show larger gaze cueing effects than those identifying themselves as male in some contexts (Bayliss et al., 2005; McCrackin & Itier, 2019). However, as we have shown in our study, a mean difference in gaze cueing magnitudes is not enough to conclude that the cognitive mechanisms underlying male and female cueing effects are different. We were unable to assess this possibility further as participant sex was not linked with each individual’s responses in all of the raw data sets. Research has also found, in some contexts, a negative association between autism-like traits and gaze cueing magnitudes (Bayliss et al., 2005), but we are unable to test this possibility given the available data. Furthermore, it is important to note that more recent investigations of this association have yielded mixed findings at best, with some studies failing to replicate the association all together (Lassalle & Itier, 2015; McCrackin & Itier, 2019; Sato et al., 2010, 2017). This variation on the individual level found in our study also calls into question whether positive gaze cueing effects actually occur for all people. We showed that some people’s gaze cueing magnitudes were negative or close to zero, but it was beyond the scope of the current study to infer whether this meant these people do not actually display gaze cueing effects rather than simply representing noise in data collection. One way that future research could assess whether there is genuine individual variability in who actually displays positive gaze cueing effects would be to use a constrained Bayesian mixed-modelling approach as described by Haaf and Rouder (2017).

A natural question that arises from our study is whether the results are exclusive to gaze cues or whether we would expect the same kind of results for arrow cues or other equivalent non-biological cueing paradigms. Indeed, this question as to whether gaze cues elicit unique cueing effects has been of substantial interest within the gaze cueing literature (Caruana et al., 2015; Chacón-Candia et al., 2023; Chen et al., 2022; Ha & Hayward, 2023; Hermens & Walker, 2010; Hietanen et al., 2008; Joseph et al., 2015; Kuhn & Kingstone, 2009; Marotta et al., 2019; Singh et al., 2022; Stevens et al., 2008; Tipper et al., 2008; Tipples, 2008), yet conflicting results continue to emerge. In Supplementary Material 12, we analysed arrow cueing data from participants in Data Set 1 (as those participants were exposed to arrow cues and gaze cues). Overall, we found that the arrow cues from Data Set 1 elicit very similar responses compared with the gaze-cues from the same data set, such that non-decision time was the parameter most likely to be underlying arrow cues when collapsing across participants. However, also similar to the gaze cues in Data Set 1, among the arrow cueing data there was a high proportion of participants who were best described by the null model, and the relative confidence for the best performing models was fairly low, particularly compared with Data Sets 2 and 3. This noise in the data is not unexpected given the relatively small number of participants and trials per participant, and it could be why on neither gaze nor arrow cued responses in Data Set 1 do we observe the reliable individual differences for starting point. There was also low internal consistency within participants’ comparative responses to gaze and arrow cues, whereby many of the same participants who had large positive cueing magnitudes for arrows, had cueing magnitudes that were very small or negative for gaze. Of course, this could suggest that some people respond differently to gaze and arrow cues, but given the problems with this data set that we have already discussed, we are reluctant to make any strong inferences.

While it is important to be clear that we do not have sufficient data to make any particular claims about arrow cueing from our data, it is possible that the presence of the individual differences in starting point could indicate a unique effect of gaze cued faces. Resource allocation models of attention tend to perform best in tasks that involve more complicated processing (such as near-threshold target discrimination tasks, or tasks with perceptual noise or distractors) compared with the localisation tasks used in the current study (Sewell & Smith, 2012; Voss et al., 2004), suggesting the gaze cues could, in theory, be prompting the increased allocation of information processing resources for some participants. In support of this idea, some social-cognitive research suggests that people allocate more information processing resources to jointly attended locations (Reid et al., 2004; Reid & Striano, 2005; Shteynberg, 2015; Wahl et al., 2013, 2019), and individual differences in facial processing are widely documented (Wilmer, 2017), so it is possible that when analysing a more robust data set of equivalent non-biological cues, we might not see these individual differences in attentional orienting versus resource allocation mechanisms, and instead only observe attentional orienting mechanisms for non-biological cues. In the current study we can only speculate about this possibility, but it highlights how evidence accumulation models could be used to differentiate gaze cues from non-biological cues in future research.

We found by far the least support for a drift rate account, suggesting that it is very unlikely that gaze cues have a sustained influence on information processing such that people allocate more information processing resources to the gazed-at location for the duration of the evidence accumulation process. These results contrast with the only other study applying evidence accumulation modelling to the gaze cueing effect by Parker and Ramsey (2022a), which suggested that drift rate was responsible for the gaze cueing effects observed in their study. Critically, however, in their study drift rate was the only mechanism that was allowed to vary across cued and miscued trials, meaning that it was impossible for them to identify whether any other mechanism was driving the effect. Our results suggest that had they allowed starting point or non-decision time to vary across cued and miscued trials, the effect of drift rate may be substantially reduced (although it is important to note that they implemented a more complicated gaze cueing paradigm to what we used in our study). This lack of a drift rate effect found in our study qualifies the generalisability of previous finding that suggest suggest people recruit greater cognitive resources when another person is looking at the same object (Reid et al., 2004; Reid & Striano, 2005; Shteynberg, 2015; Wahl et al., 2013, 2019). Specifically, according to our study, most localisation gaze cueing effects are not driven by increased allocation of information processing resources to the gazed-at location and for the minority where this is the case, this shift in resource allocation is very short and dissipates over time. However, it is possible that the localisation paradigms we used were too simple to necessitate this reallocation of information processing resources. In more difficult gaze cueing paradigms that require more information processing to initiate a correct response, such as those requiring a target-categorisation response, it is plausible that an information processing mechanism could have a stronger influence on gaze cueing effects. Future studies should apply the models used here to other kinds of gaze cueing paradigms that require more information processing resources, to see whether they generate different cognitive responses.

An important limitation of our design was that we ignored the distinction between several potential moderating factors present in the data sets used, such as cue emotional expression and cue–target SOA. The presence of these moderating factors in the data could potentially explain why there appeared to be some noise in the data sets or why there were differences between data sets more broadly, since many of these moderating factors did attenuate cueing effects in the original studies (Carlson, 2016; Chen et al., 2021; Gregory & Jackson, 2020). One of the reasons we did not investigate moderating factors in our study was in part because there were not enough trials per person and per cell in the original studies to allow for reliable parameter estimates of each moderating factor (Evans, 2020; Lerche et al., 2017). The only data set which had sufficient trials per participant and per condition to look at moderating factors was Data Set 3, though there did not appear to be any obvious qualitative differences in subserving mechanisms as a function of those moderating factors (SOA and emotional expression; see Supplementary Material 7). Although this requirement of a large number of trials per person could be considered a more general constraint of evidence accumulation models, we believe that the advantages of an evidence accumulation modelling approach far outweigh the costs of needing to collect more data per person (which would usually be beneficial to the reliability of any results anyway). Further, we have demonstrated in our study that it is important to investigate the gaze cueing effect at the individual level, which necessitates a substantial number of trials per person even without an evidence accumulation modelling approach (Rouder et al., 2019; Smith & Little, 2018). We encourage future research to use evidence accumulation models to investigate whether different moderating factors influence people’s underlying cognitive responses to gaze cues.

Although we believe the DDM was an appropriate initial model to begin understanding the gaze cuieng effect within a computational modelling framework due to its simplicity and common use, future research should consider whether other types of models provide a different insight into the mechanisms underlying the gaze cueing effect. While the DDM did a reasonable job accounting for the data, it was limited in its ability to describe some trends, such as the tendency for cueing effects to reverse at later stages of the response time distributions. One logical next step for future research would be to assess whether models of conflict tasks, such as the conflict diffusion models (Evans & Servant, 2020; Hübner et al., 2010; Lee & Sewell, 2023; Ulrich et al., 2015; White et al., 2011) are better able to capture the patterns of data in the gaze cueing task. Conflict evidence accumulation models hold many of the same assumptions as standard evidence accumulation models, but have been designed to assess the influence of task-irrelevant information, such as the type of distractors found in Stroop or Flanker tasks. These models would therefore be able to assess the degree to which people are trying to suppress gaze cues and disregard them as task-irrelevant information. Conflict models have also been found to be able to account for the ordering of response times between conditions to overlap or reverse later in the response time distribution (Lee & Sewell, 2023; Ulrich et al., 2015), which suggests they could be useful for further understanding the gaze cueing effect in future investigations. We would like to emphasise, however, that although assessing other models in gaze cueing task is a useful future direction, we believe that the DDM was an appropriate first model to use.

Keeping in mind the various constraints discussed here, we posit that our study suggests three main takeaways. First, the process that underlies the majority of people’s localisation gaze cueing effects is most likely to be an attentional orienting process. However, second, there are individual differences such that not everyone’s gaze cueing effects are best described by that process. Third, localisation gaze cueing effects are probably not the result of a sustained shift in information processing resources to the cued location. Above all, we hope that we have shown the value of applying computational models to cueing paradigms and studies of social cognition more broadly. We have demonstrated that evidence accumulation models in particular provide a theoretically grounded method for going beyond the observed behaviour to make inferences about underlying cognitive mechanisms driving an effect. We hope that our study promotes further application of these models to gaze cueing research in future.

## Supplemental Material

sj-pdf-1-qjp-10.1177_17470218231181238 – Supplemental material for Uncovering the cognitive mechanisms underlying the gaze cueing effect Supplemental material, sj-pdf-1-qjp-10.1177_17470218231181238 for Uncovering the cognitive mechanisms underlying the gaze cueing effect by Manikya Alister, Kate T McKay, David K Sewell and Nathan J Evans in Quarterly Journal of Experimental Psychology

## Notes

1 Although non-decision time can also represent post-decision motor response, in gaze cueing paradigms we suggest that the most plausible explanation for the cueing paradigms used in this study is that non-decision time would represent attentional selection, which is supported by research showing that gaze cues do not induce automatic motor-response activation (Vainio et al., 2014).

2 Note that a more accurate representation of these more complex processing dynamics would be a two-phase piecewise model, where the first phase involves a substantially higher drift rate that is based on the cueing information, and the second phase involves regular processing of the target (e.g., similar to the model of Hübner et al., 2010). However, due to the computational difficulties associated with piecewise models (Evans, Trueblood, & Holmes, 2020; Holmes et al., 2016), we restrict our modelling assessments to the standard diffusion model framework.

3 Including between trial variability is much more computationally intensive and has been shown to cause parameter identifiability issues and other problems with parameter estimation (see Boehm et al., 2018; Evans, Tillman, & Wagenmakers, 2020; Lerche & Voss, 2016; van Ravenzwaaij & Oberauer, 2009). However, we repeated a number of the individual-level analyses reported here with the diffusion decision model (DDM) that included between-trial variability parameters for drift rate, starting point, and non-decision time, but found that model fits and model comparisons were equivalent, but with more individual variability in the model comparisons. See Supplementary Materials 8–11.

4 We analyse the arrow cues in Supplementary Material 12.

5 We were unable to discern the sex or gender breakdown of participants subject to analysis in Data Set 3 as neither participant sex nor gender was available in the raw data.

6 We performed a recovery analysis to ensure that parameters could be reliably identified in this model. See Supplementary Material 2 for more details.

7 These constraints on the individual level differed slightly from the pre-registered analysis plan. Originally, we had planned to completely constrain the individual-level estimates, but after running the analyses we realised that this did not permit sufficient flexibility, resulting in differences in parameter estimates which looked reliable but were most likely artefacts of the model constraints. Specifically, the previous results showed a highly reliable but extremely small z change, which was most likely caused by the fact that we were forcing the individual-level estimates to move in that direction. We discuss this more and show the results of the original pre-registered analysis in Supplementary Material 4.
