# Supplementary material for: Uncovering the cognitive mechanisms underlying the gaze cueing effect
Source: Q J Exp Psychol (Hove). 2023 Jun 27;77(4):803–27. doi: 10.1177/17470218231181238 (PMC10960327; doi:10.1177/17470218231181238)
Supplement: sj-pdf-1-qjp-10.1177_17470218231181238 – Supplemental material for Uncovering the cognitive mechanisms underlying the gaze cueing effect [file sj-pdf-1-qjp-10.1177_17470218231181238.pdf]

Uncovering the Cognitive Mechanisms Underlying the Gaze Cueing Effect

Supplementary Materials

Contents

Supplementary Material 1: Priors for the Individual-Level Modelling **4**

Supplementary Material 2: DDM Recovery Analysis **5**

Supplementary Material 3: Priors for the Hierarchical Modelling **6**

Supplementary Material 4: Results of the Originally Preregistered Hierarchical Modelling Analysis **7**

Supplementary Material 5: Individual-Level Modelling Fits **9**

Supplementary Material 6: AIC Inclusion Probabilities **13**

Supplementary Material 7: Qualitative Assessment of Moderating Factors in Data Set 3 **14**

Supplementary Material 8: Individual-Level Analysis with DDM Including Between Trial Variability Parameters **17**

Data Set 1 17

Individual-Level Modelling 17

Data Set 2 19

Individual-Level Modelling 19

Data Set 3 21

Individual-Level Modelling 21

Supplementary Material 9: Recovery of DDM With Between Trial Variability Parameters **23**

Supplementary Material 10: Model Fits with Between Trial Variability Parameters **24**

Supplementary Material 11: Qualitative Assessment of Moderating Factors in Data Set 3 with Between Trial Variability Parameters **25**

Supplementary Material 12: Arrow Cueing Analysis **28**

Method 29

Results 30

Stage 1: Cueing Magnitudes 30

Stage 2: Individual-Level Modelling 30

Stage 3: Bayesian Hierarchical Modelling 32

Supplementary Material 1: Priors for the Individual-Level Modelling

Definition of terms:

μ = group-level mean

σ = group-level standard deviation

i = participant

j = cue condition (cued or miscued)

t0 = non-decision time

a = threshold

$\frac{z}{a}$ = relative (to threshold) starting point

v = drift rate

N = normal distribution

TN = truncated normal distribution

γ = gamma distribution

Prior Distributions:

$v_j \sim N(3{,}1)$

$\frac{z_j}{a} \sim TN(0.5,\ 0.1\ ,0,\ 1)$

$t0_j \sim TN(0.3,\ 0.1,\ 0,\ \infty)$

$a \sim TN(2,\ 1,\ 0,\ \infty)$

Supplementary Material 2: DDM Recovery Analysis

We performed a recovery analysis on the Diffusion Decision Model that appears in the manuscript to ensure the parameters could be reliably identified from each other. This was achieved by first simulating 100 data sets with a sample of generated parameters (sampled using Latin Hypercube Sampling), then fitting the DDM to that dataset to see whether the estimated parameters were correlated with the generated parameters. Specifically, we generated and estimated parameters for each gaze cueing condition (i.e., a parameter for cued and miscued trials) for parameters that varied across conditions, except for z since z in the miscued condition was 1− z in the cued condition. As can be seen in Table 1, we found a high correlation between estimated and generated data sets, suggesting that the parameters were identifiable.

Table 1: Correlations between estimated and generated parameters for each gaze cue condition with no between trial variability parameters.

Parameters	Correlation	Generating Ranges (Upper, Lower)
z	0.99	(0.1,4)
v_c	0.95	(-0.9,5)
v_m	0.94	(-0.9,5)
$t0_c$	0.97	(0,0.7)
$t0_m$	0.96	(0,0.7)
a	0.93	(0.45, 1.75)

Note. $_c$ subscript denotes the cued condition and $_m$ subscript denotes miscued condition. Generating ranges refers to the possible values that could be sampled for each generating parameter via Latin Hypercube Sampling.

Supplementary Material 3: Priors for the Hierarchical Modelling

Prior Distributions:

Individual Level:

$v_{ij} \sim N(\mu_{vj}, \sigma_v)$

$\frac{\frac{z}{a}_{ij}}{a_i} \sim TN(\mu_{\frac{z}{a}j}, \sigma_{\frac{z}{a}}, 0, 1)$

$t0_{ij} \sim TN(\mu_{t0j}, \sigma_{t0}, 0, \infty)$

$a_i \sim TN(\mu_a, \sigma_a, 0, \infty)$

Group Level:

$\mu_{vj} \sim N(3, 1)$

$\mu_{\frac{z}{a}j} \sim TN(0.5, 0.1, 0, 1)$

$\mu_{t0j} \sim TN(0.3, 0.1, 0, \infty)$

$\mu_a \sim TN(2, 1, 0, \infty)$

$\sigma_v, \sigma_a, \sigma_{t0}, \sigma_{\frac{z}{a}} \sim \gamma(1, 1)$

Supplementary Material 4: Results of the Originally Preregistered Hierarchical Modelling Analysis

As discussed in the manuscript, we preregistered a slightly different hierarchical-level analysis compared to what was included in the manuscript. Originally, we had planned to completely constrain the individual-level estimates, but after running the analyses we realized that this did not permit sufficient flexibility, resulting in differences in parameter estimates which looked reliable but were most likely artifacts of the model constraints, as shown in Figure SM.1 and discussed in detail below.

Figure SM.1.

Differences in Group Level Parameter Estimates Across Cued and Miscued Trials

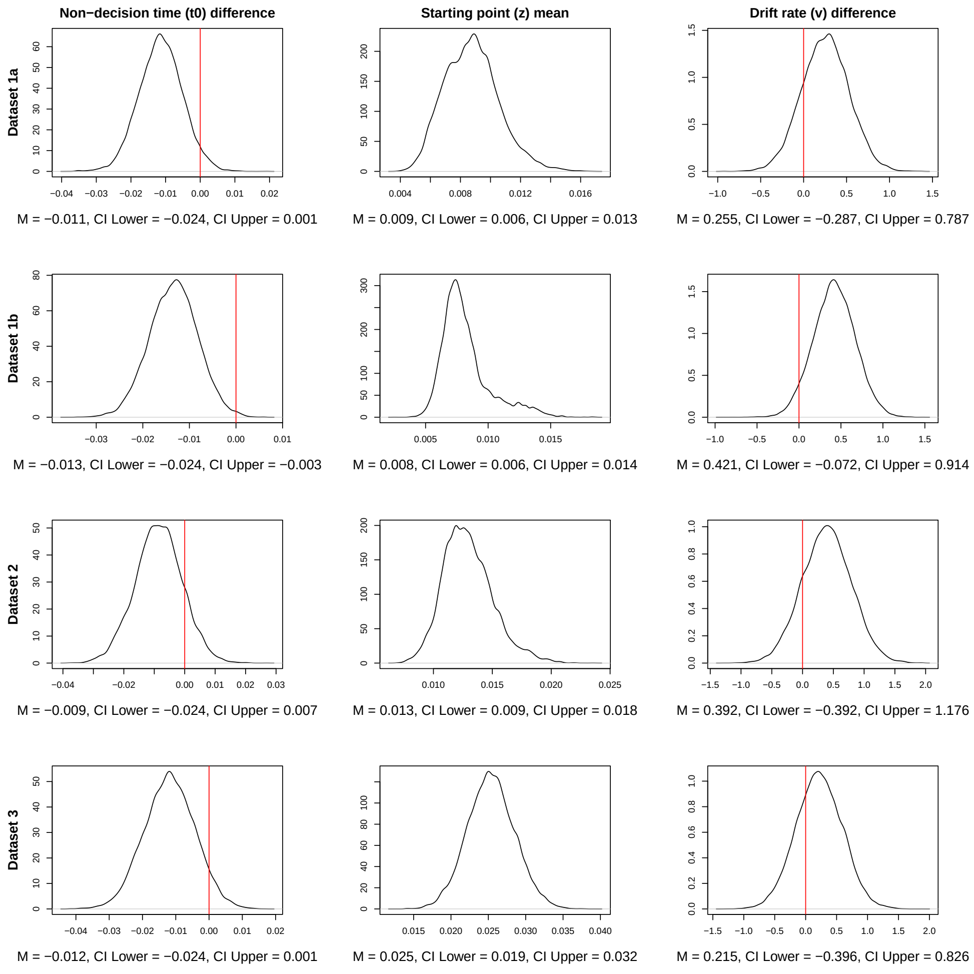

As is shown in Figure SM.1, constraining the individual-level of the hierarchical analyses resulted in too much credibility being allocated to z, whereby z appeared to show highly credible – yet extremely small – shifts across cued and miscued trials. We think it is unlikely that these results reflect true shifts in z across trials but rather, are an artifact of the parameter restraints given that none of the parameters were permitted to move in a theoretically implausible direction at the individual level. We believe this to be the case because this seemingly credible yet extremely small differences in z were not maintained when we unconstrained the individual level estimate for z while constraining the other parameters (as shown in the analyses presented in the manuscript). The estimates of t0, however, did remain fairly stable in comparison.

Further, the results obtained in the original analyses (shown in Figure **??**) seem less plausible due to their inconsistency with the individual-level modelling, which did not find a high probability that z was subserving cueing effects. Moreover, the results for z in the analyses below seem consistent with extreme hierarchical shrinkage, where many individuals with parameter values in one area (i.e., right on 0, as the parameter values of individuals are not allowed to be below zero in this analysis) strongly pull the values of other individuals towards them, resulting in a highly certain group-level estimate due to the tight clustering of the shrunken individual parameter estimates.

Supplementary Material 5: Individual-Level Modelling Fits

This supplementary material shows the predictions of each individual-level model for the models presented in the main manuscript plotted against each data set. These plots show the mean observed and predicted response times (x axes) for each quantile of the response time distribution (y axes) (see figures SM.2, SM.3, SM.4). We provide a verbal description and qualitative assessment of each of the fits within the manuscript.

Figure SM.2.

Data Set 1a Individual-Level Model Fits

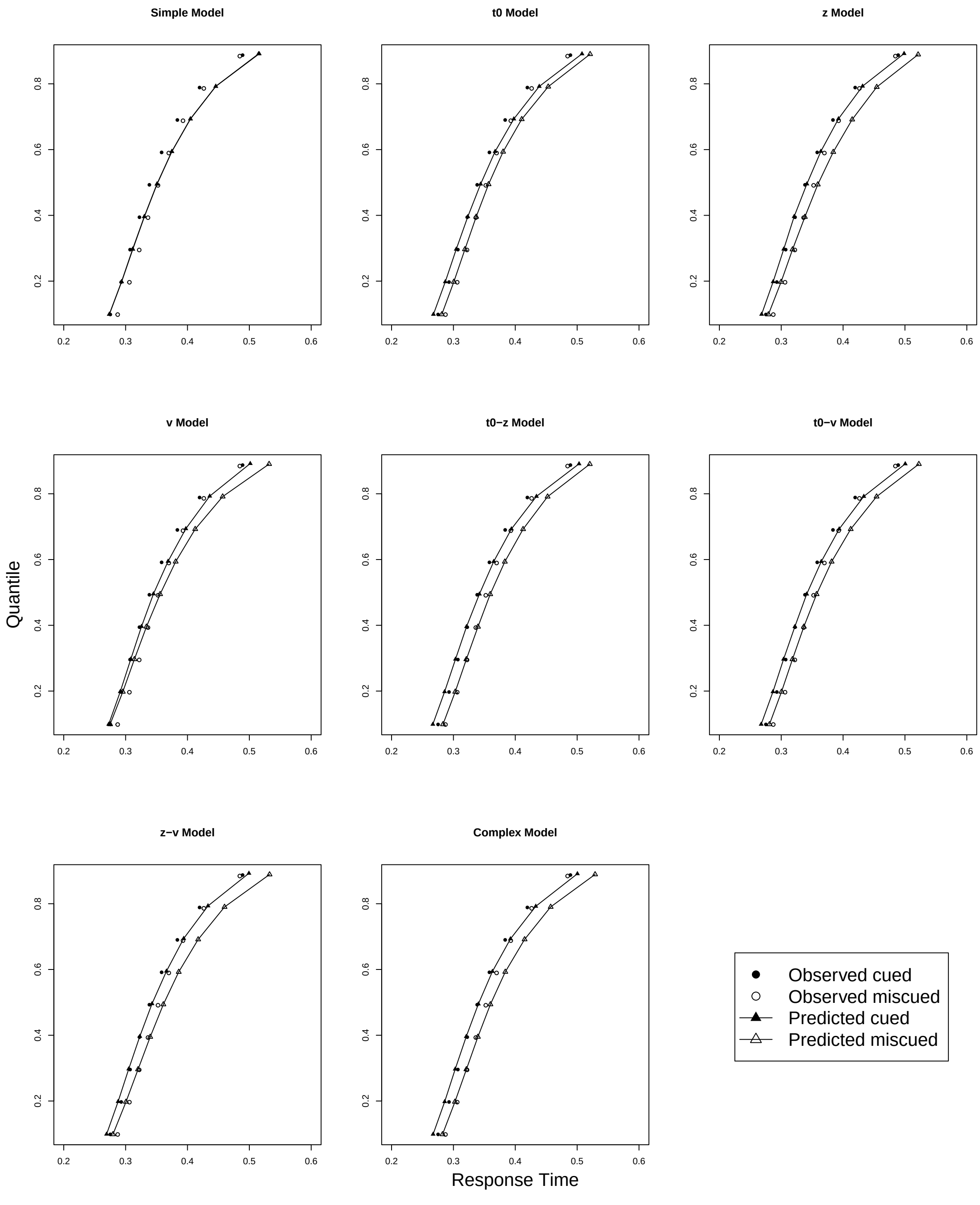

Figure SM.3.

Data Set 2 Individual-Level Model Fits

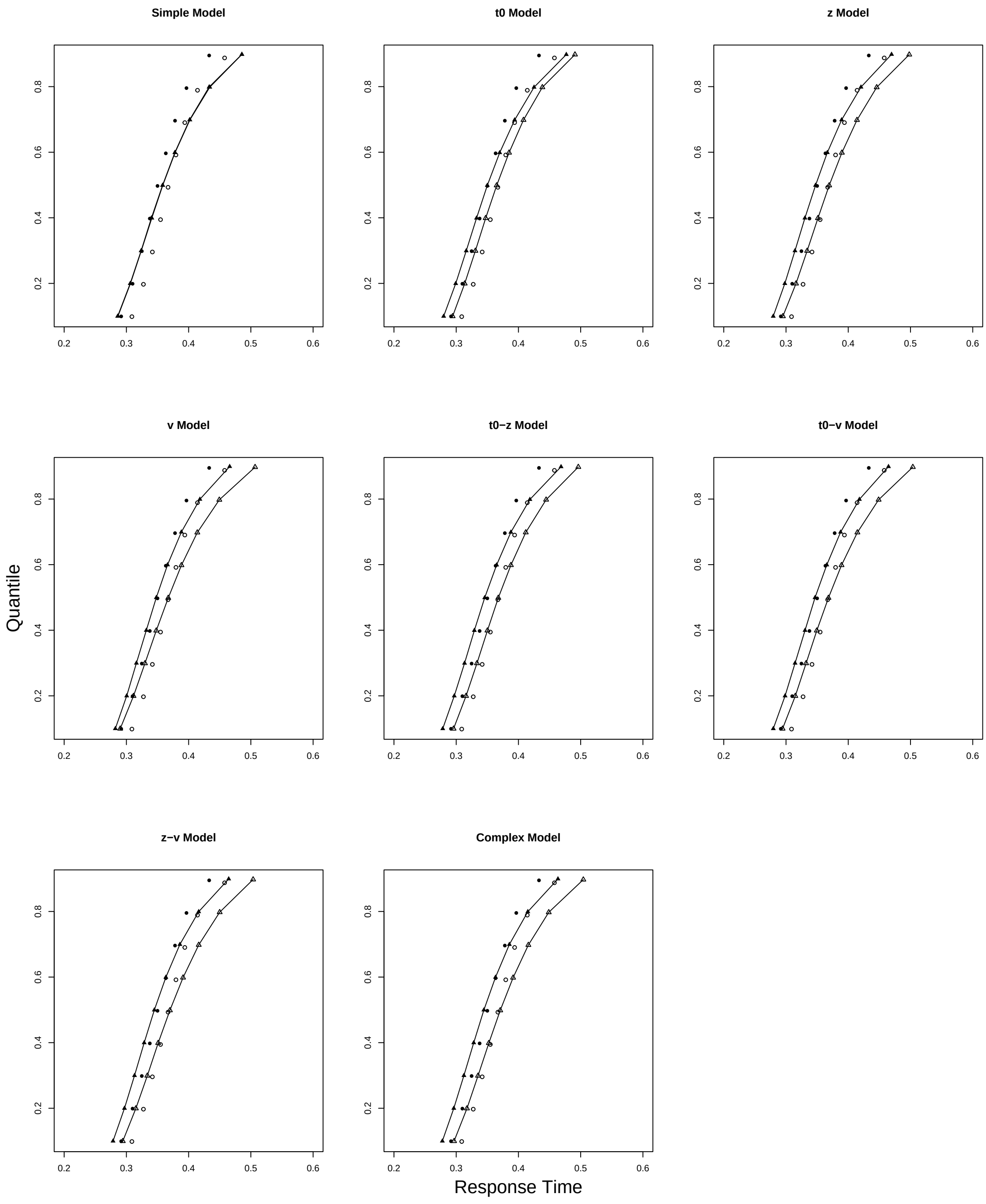

Figure SM.4.

Data Set 3 Individual-Level Model Fits

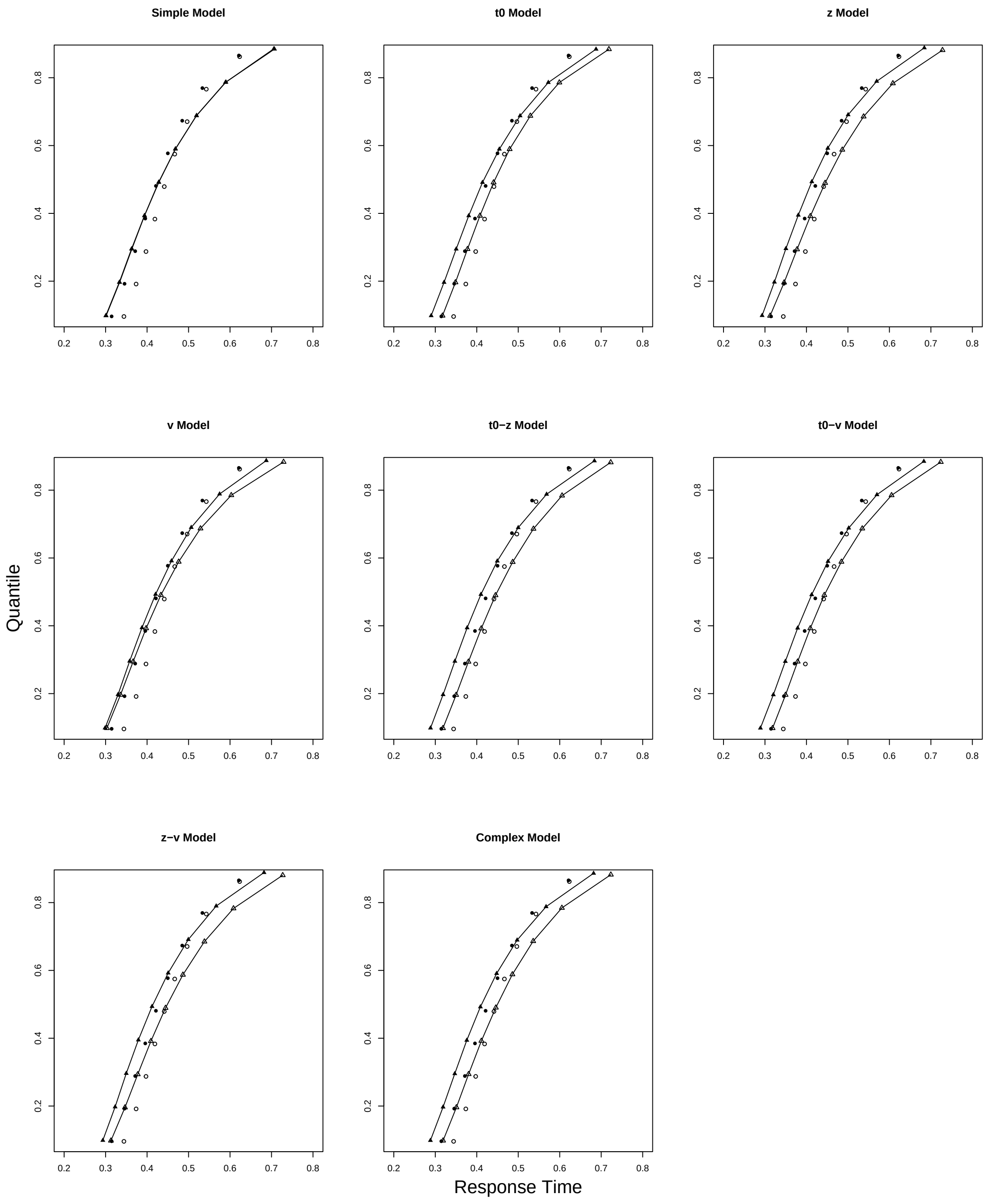

Supplementary Material 6: AIC Inclusion Probabilities

As can be seen in SM.5, AIC inclusion probabilities result in similar qualitative conclusions for each dataset to the BIC inclusion probabilities presented in the manuscript. As discussed in the manuscript, the main difference between AIC and BIC is that *all* of the models are relatively more likely compared to null models in BIC, as BIC penalises flexibility more than AIC.

Figure SM.5.

AIC Inclusion Probabilities.

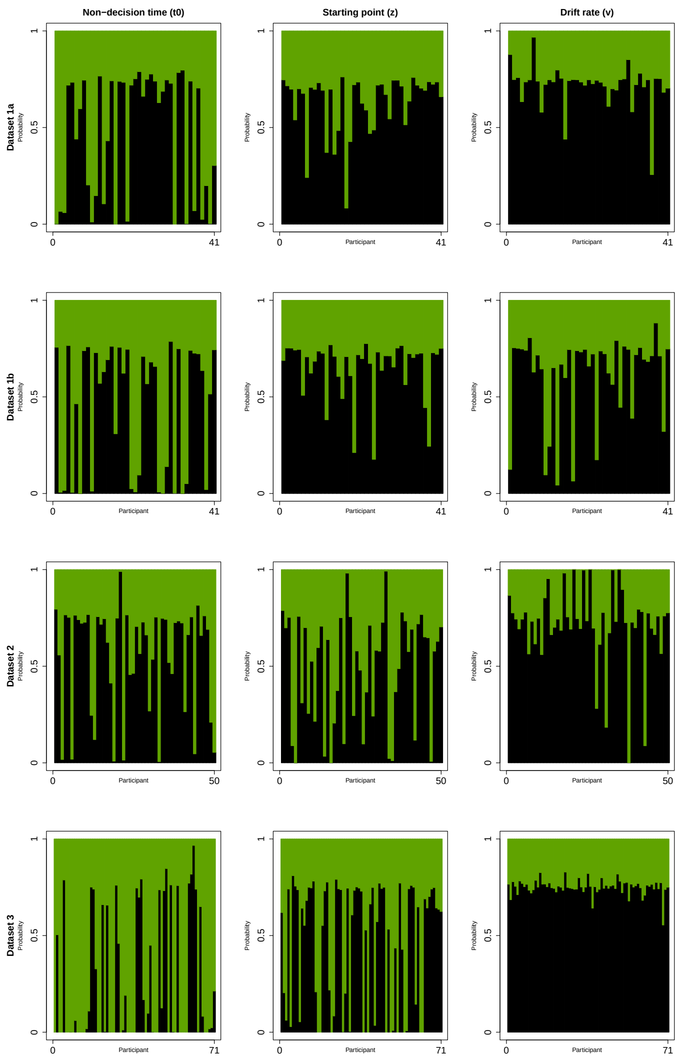

Figure SM.6. *Note.* Green represents models that assume the respective parameter varies across conditions. Larger proportion of green in these figures indicates broader support for a parameter driving differences across cued and miscued conditions. See the manuscript for a more detailed description of this analysis.

Supplementary Material 7: Qualitative Assessment of Moderating Factors in Data Set 3

As mentioned in the Discussion of the main manuscript, a limitation of our design was that we ignored several potential moderating factors in the original data sets. One of the main reasons why we did not assess these potential moderating factors was because in most cases, there were not enough trials to reliably estimate DDM parameters for these moderating factors. This was, however, possible for Data Set 3. In this supplementary material, we assessed each potential moderating factor that was present in Data Set 3: SOA (200ms, 500ms) and Cue Emotional Expression (Happy, Fearful, Neutral, Angry), as separate data sets to see whether our results changed as a function of these moderating factors.

As shown in Figure SM.7 and Figure SM.8, there did not appear to be any obvious qualitative differences across moderating factors. We see a higher probability for the simple model in most plots compared to those reported in the manuscript, but this is because there were fewer trials and, particularly BIC, will favor simpler models when there are fewer trials. Therefore, we think it is reasonable to suggest that the overall conclusions from out paper hold across these SOA and cue emotion manipulations, at least for this data set.

Figure SM.7.

Weighted BIC Model Probabilities as a Function of Moderating Factor.

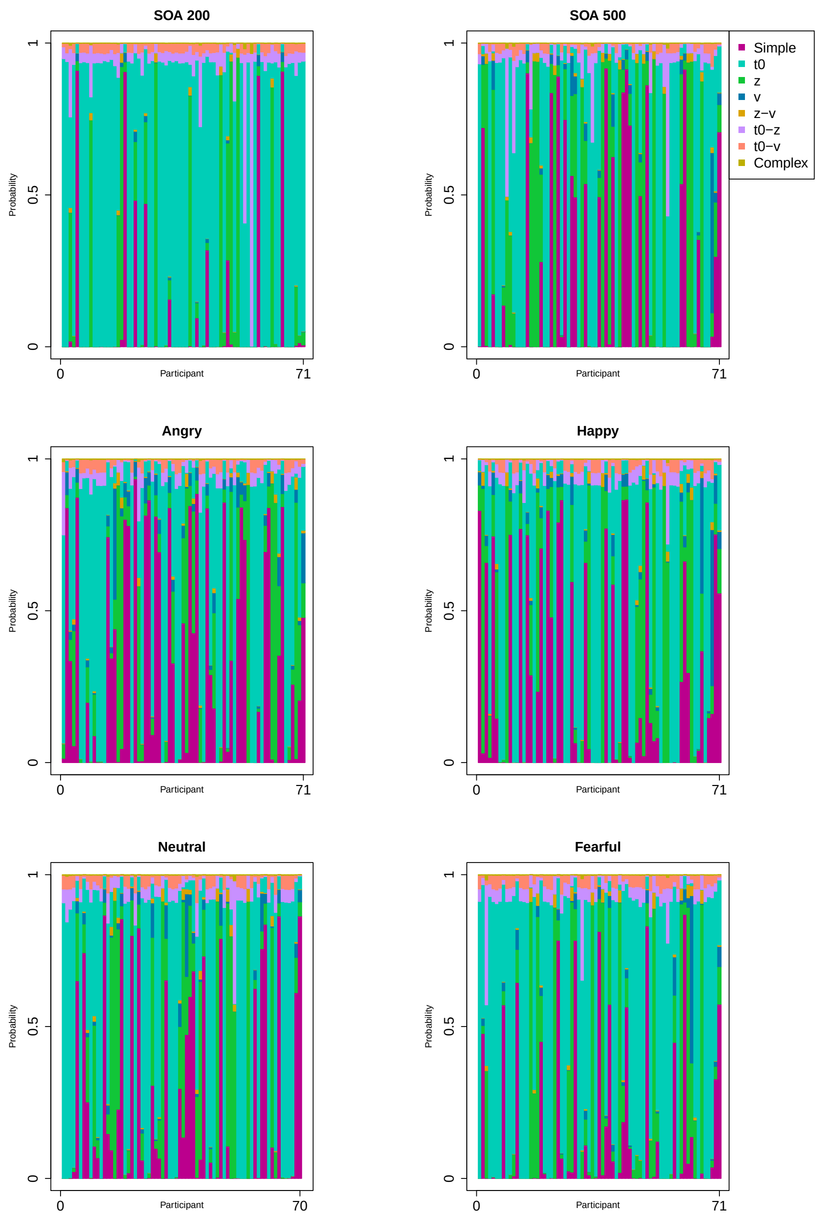

Figure SM.8.

Weighted BIC Model Probabilities as a Function of Moderating Factor.

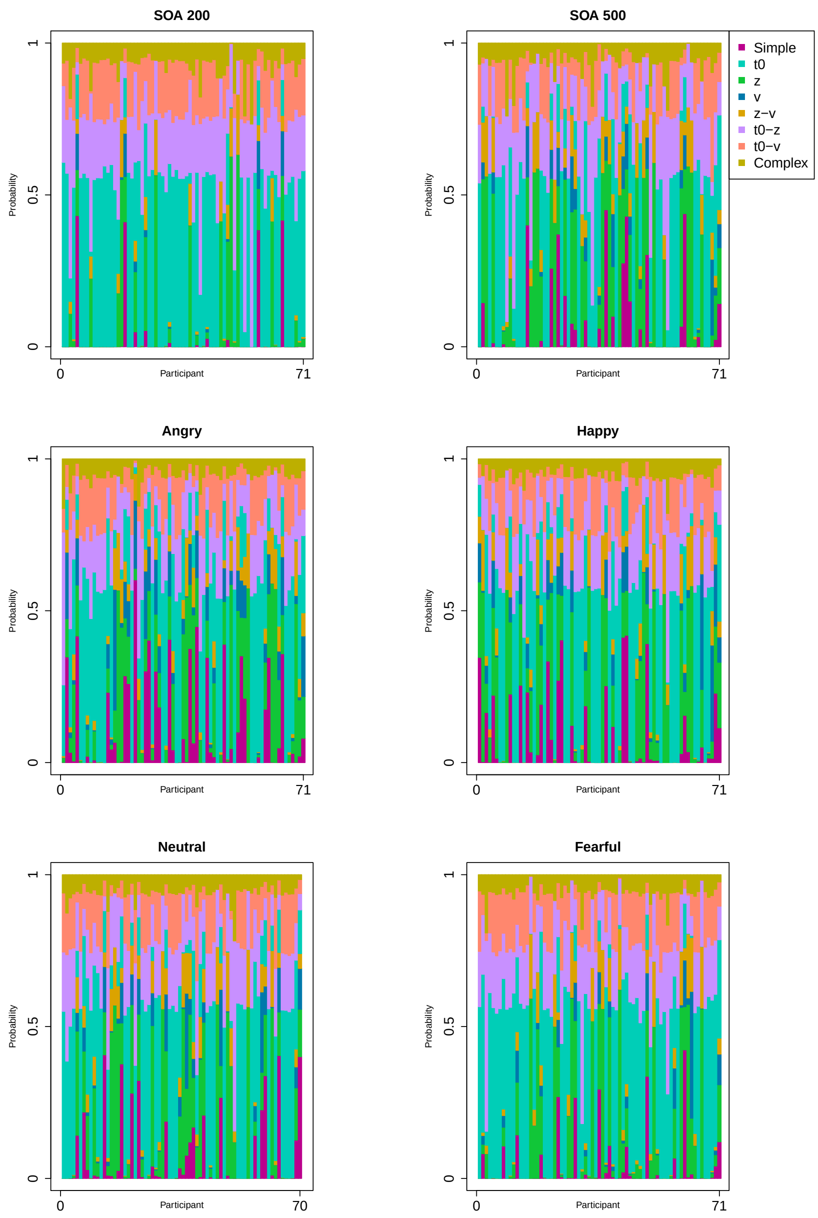

Table 2: Model performance as indicated by the individual-level modelling for Data Set 1 collapsed across participants. "Probability" represents the relative probability of each model being the best candidate model according to BIC and AIC respectively. "Raw Score" corresponds to the raw BIC and AIC scores.

Model	BIC		AIC	
	Probability (%)	Raw Score	Probability (%)	Raw Score
Simple	40	-519.33	16	-540.21
t0	33	-520.77	31	-545.12
z	15	-519.21	20	-543.57
v	8	-512.84	5	-533.37
v-z	1	-512.56	5	-540.39
t0-v	1	-514.62	8	-542.45
t0-z	2	-515.73	12	-543.56
Complex	0	-509.36	3	-540.67

Note. Higher probabilities indicate a higher probability of that model being the best performing model. Lower raw BIC and AIC scores indicate better model performance. To get the values shown in this table, we calculated a unique raw AIC and BIC and weighted AIC and BIC for each participant and model, and then calculated the average values by collapsing these individual scores across participants.

Supplementary Material 8: Individual-Level Analysis with DDM Including Between Trial Variability Parameters

In this section, we replicated the individual-level analyses presented in the manuscript with a version of the DDM that included between trial variability parameters. There did not appear to be any obvious qualitative differences between the two analyses, although the z model performed better in some participants.

Data Set 1

Individual-Level Modelling

Weighted Model Probabilities The top panel of figure SM.9 shows a graphical representation of the probability that each model is the best performing model for each participant in Data Set 1 according to BIC and AIC. Table2 shows the raw BIC and AIC values collapsed across participants as well as the exact weighted BIC and AIC probabilities collapsed across participants.

According to BIC as shown in Figure SM.9, 20 participants were best described by the simple model, 14 participants were best described by the t0 model, and 5 by the z model. When collapsing across participants and taking the mean, as shown in Table 2, according to BIC the Simple model had the highest probability of being the best performing model, followed by the t0 model, although t0 still had the best raw BIC score.

Figure SM.9. BIC and AIC Weighted Model Probabilities for Each Participant and Data Set Using the Diffusion Model with Between Trial Variability Parameters

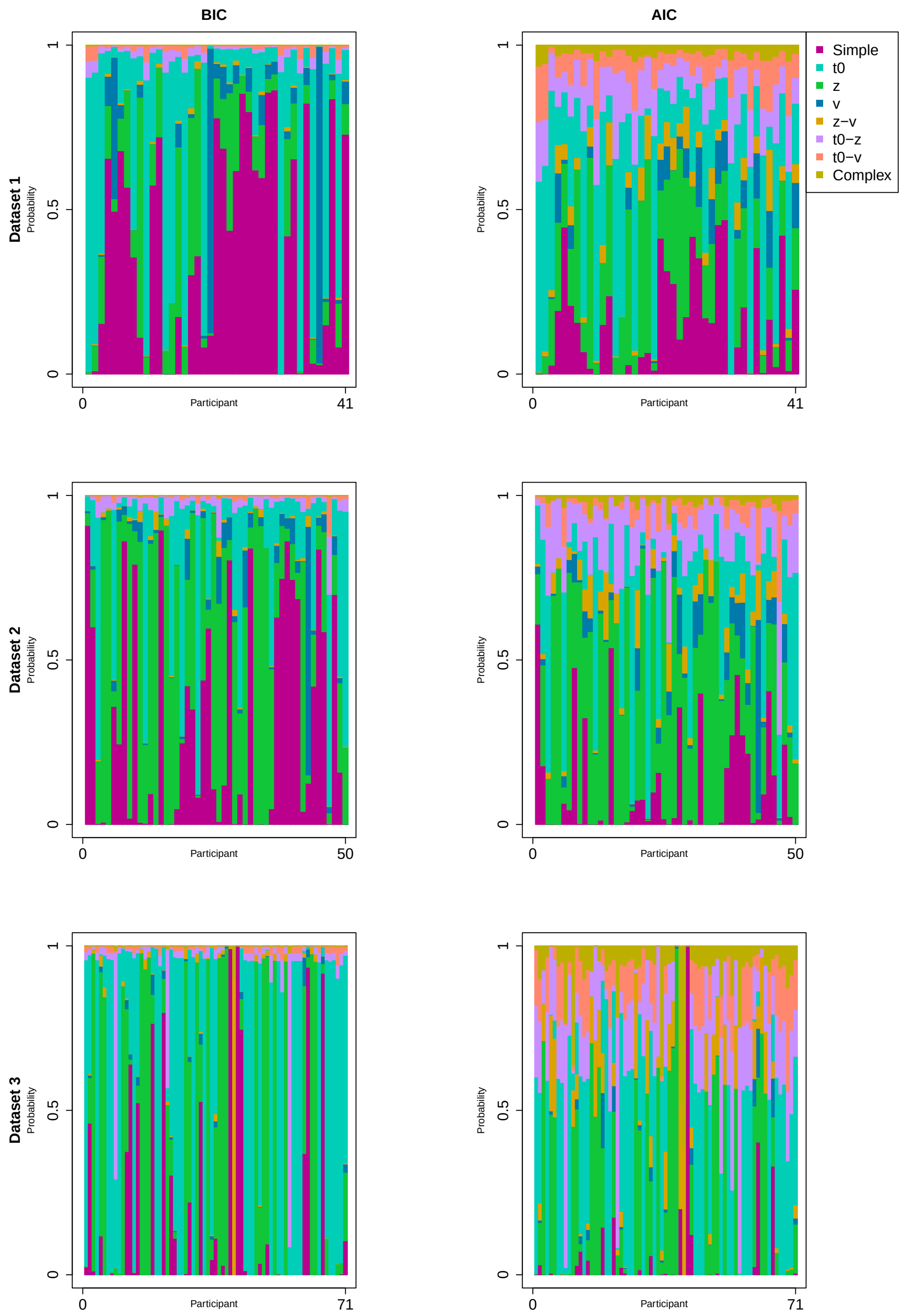

According to AIC, 17 participants were best described by the t0 model, 13 by the simple model, 10 by the z model, and 1 by the v-z model. When collapsing across participants, as shown in Table 2, according to AIC, the t0 model was most likely to be the best performing candidate model followed by the simple model. These individual-level results as a whole appear to be qualitatively equivalent to those reported in the manuscript.

Parameter Inclusion Probabilities As shown in the top panel of Figure SM.10, 14 participants had greater than 50% chance of being best described by models that permitted t0 to vary across conditions. Two participants had greater than 50% chance of being best described by models that permitted z to vary across conditions. Zero participants had a greater than 50% chance of being best described by models that permitted v to vary across conditions.

Collapsing across participants, models that assumed t0 varied across cued and miscued trials were on average 36% likely to be the best candidate model when compared to models that did not make this assumption (z, v, z-v, simple; see Figure SM.10, top panel). Models that assumed z varied across cued and miscued trials were on average 18% likely to be the best candidate model compared to models that did not make this assumption. Models that assumed v varied across cued and miscued trials were on average 10% likely to be the best candidate model compared to models that did not make this assumption.

Data Set 2

Individual-Level Modelling

Weighted Model Probabilities According to BIC and as shown in Figure SM.9, 18 participants were best described by the simple model, 18 by the z model, 12 by the t0 model, and 3 by the v model. As shown in Table 4, averaged across participants, the z model was the most likely to be the best performing candidate model followed by the simple model. According to AIC, 22 participants were best described by the z model, 15 by the t0 model, 3 by the v model, and 1 by the t0-z model. Averaging across participants, the z model was the most likely to be the best-performing candidate model followed by the t0 model.

Parameter Inclusion Probabilities As shown in the third panel of Figure SM.10, 11 participants had a greater than 50% chance of being best described by models that permitted t0 to vary across conditions. Fifteen participants had a greater than 50% chance of being best described by models that permitted z to vary across conditions. Two participants had a greater than 50% chance

Figure SM.10. BIC Parameter Inclusion Probabilities for Each Participant and Data Set Using the Diffusion Model with Between Trial Variability Parameters

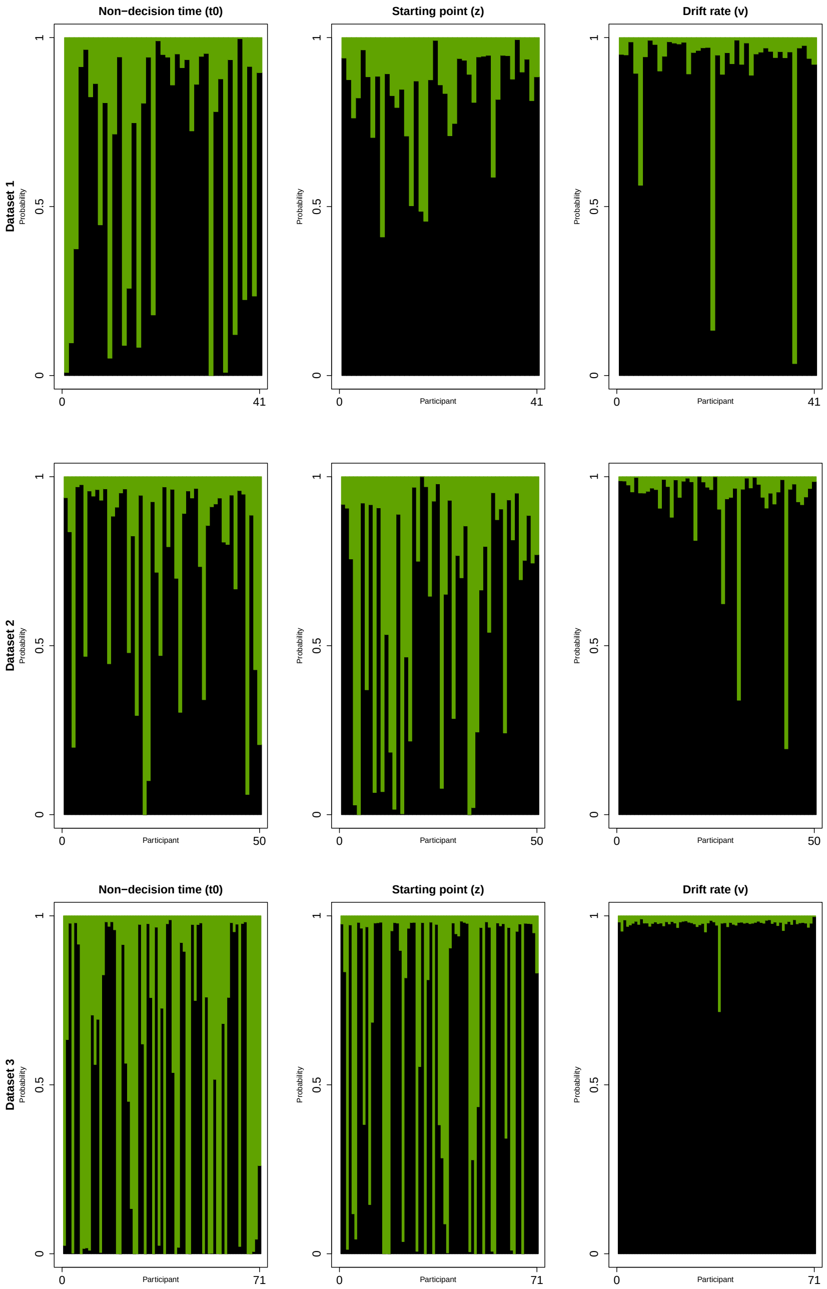

Note. Green represents models that assume the respective parameter varies across conditions. Larger proportion of green in these figures indicates broader support for a parameter driving differences across cued and miscued conditions.

of being best described by models that permitted v to vary across conditions.

Collapsing across participants, models that assumed t0 varied across cued and miscued trials were on average 17% likely to be the best candidate model when compared to models that did not make this assumption. Models that assumed z would vary across cued and miscued trials were on

Table 3: Model performance as indicated by the individual-level modelling for Data Set 2 collapsed across participants. "Probability" represents the relative probability of each model being the best candidate model according to BIC and AIC respectively. "Raw Score" corresponds to the raw BIC and AIC scores.

Model	BIC		AIC	
	Probability (%)	Raw Score	Probability (%)	Raw Score
Simple	31	-637.58	11	-658.84
t0	23	-639.60	24	-664.41
z	36	-642.37	34	-667.18
v	6	-636.42	7	-661.23
v-z	1	-634.63	5	-662.98
t0-v	1	-633.40	4	-661.76
t0-z	2	-637.05	12	-665.40
Complex	0	-629.54	2	-661.43

Note. Higher probabilities indicate a higher probability of that model being the best-performing model. Lower raw BIC and AIC scores indicate better model performance.

average 30% likely to be the best candidate model compared to models that did not make this assumption. Models that assumed v varied across cued and miscued trials were 3% likely to be the best candidate model compared to models that did not make this assumption. These results largely align with the results reported in the main manuscript, but with slightly more evidence in favour of z.

Data Set 3

Individual-Level Modelling

Weighted Model Probabilities According to BIC, as shown in Figure SM.9, 31 participants were best described by the t0 model, 25 by the z model, 12 by the simple model, 3 by the t0-z model, and one by the z-v model. As shown in Table 4, averaged across participants, the t0 model was the most likely to be the best performing model followed by the z model. According to AIC, as shown in Figure SM.9, 39 participants were best described by the t0 model, 22 by the z model, 7 by the z-t0 model, and 3 by the simple model. As shown in 4, averaged across participants, the t0 model had the highest probability of being the best performing model followed by the z model.

Parameter Inclusion Probabilities As shown in the third panel of Figure SM.10, 30 participants had a greater than 50% chance of being best described by models that permitted t0 to vary across conditions. 24 participants had a greater than 50% chance of being best described by models that permitted z to vary across conditions. No participants had a greater than 50% chance of being best described by models that permitted v to vary across conditions.

Table 4: Model performance as indicated by the individual-level modelling for Data Set 3 collapsed across participants. "Probability" represents the relative probability of each model being the best candidate model according to BIC and AIC respectively. "Raw Score" corresponds to the raw BIC and AIC scores.

Model	BIC		AIC	
	Probability (%)	Raw Score	Probability (%)	Raw Score
Simple	15	-1766.34	3	-1798.21
t0	45	-1785.09	32	-1822.28
z	31	-1782.60	20	-1819.79
v	1	-1763.49	1	-1800.68
v-z	1	-1774.67	6	-1817.17
t0-v	1	-1799.98	10	-1842.48
t0-z	7	-1805.69	23	-1848.19
Complex	0	-1775.93	6	-1823.75

Note. Higher probabilities indicate a higher probability of that model being the best performing model. Lower raw BIC and AIC scores indicate better model performance.

Collapsing across participants, models that assumed t0 varied across cued and miscued trials were on average 53% likely to be the best candidate model when compared to models that did not make this assumption. Models assuming that z would vary across cued and miscued trials were on average 38% likely to be the best candidate model compared to models that did not make this assumption. Models that assumed v varied across cues and miscued trials were 3% likely to be the best candidate model compared to models that did not make this assumption.

Again, there did not appear to be any obvious qualitative differences between the individual-level analyses for the full diffusion model reported here and the diffusion model reported in the manuscript, although there appeared to be more participants best fit by the z model.

Supplementary Material 9: Recovery of DDM With Between Trial Variability Parameters

For consistency, and to check the reliability of the individual modeling results reported above, we performed a recovery analysis of the DDM with between trial variability of drift rate (sv), starting point (sz), and non-decision time (st0). The method was identical to Supplementary Material 2, but with the added parameters. As shown in Table 5, the inclusion of sv, s0, and st0 seemed to result in slightly lower correlations between estimated and generated z, v, and t0 – but the correlations were still very high. sv, t0, and sz themselves were not particularly identifiable, but this is not problematic as we were not making inferences on those parameters.

Table 5: Correlations between estimated and generated parameters for each gaze cue condition with between trial variability parameters.

Parameters	Correlation	Generating Ranges (Upper, Lower)
z	0.99	(0.1,4)
v_c	0.95	(-0.9,5)
v_m	0.94	(-0.9,5)
$t0_c$	0.97	(0,0.7)
$t0_m$	0.96	(0,0.7)
a	0.93	(0.45, 1.75)
sv	0.68	(0, 1.5)
sz	0.40	(0, 0.5)
$st0$	0.78	(0, 0.5)

Note. $_c$ subscript denotes the cued condition and $_m$ subscript denotes miscued condition. Generating ranges refers to the possible values that could be sampled for each generating parameter via Latin Hypercube Sampling.

Supplementary Material 10: Model Fits with Between Trial Variability Parameters

This section shows the model fits for each data set with the DDM that included between trial variability parameters (as reported in Supplementary Material 8). As shown in Figures SM.11, SM.12, and SM.13, the inclusion of between trial variability parameters did not appear to improve the model fit.

Figure SM.11.

Data Set 1 Individual-Level Model Fits With Between Trial Variability Parameters

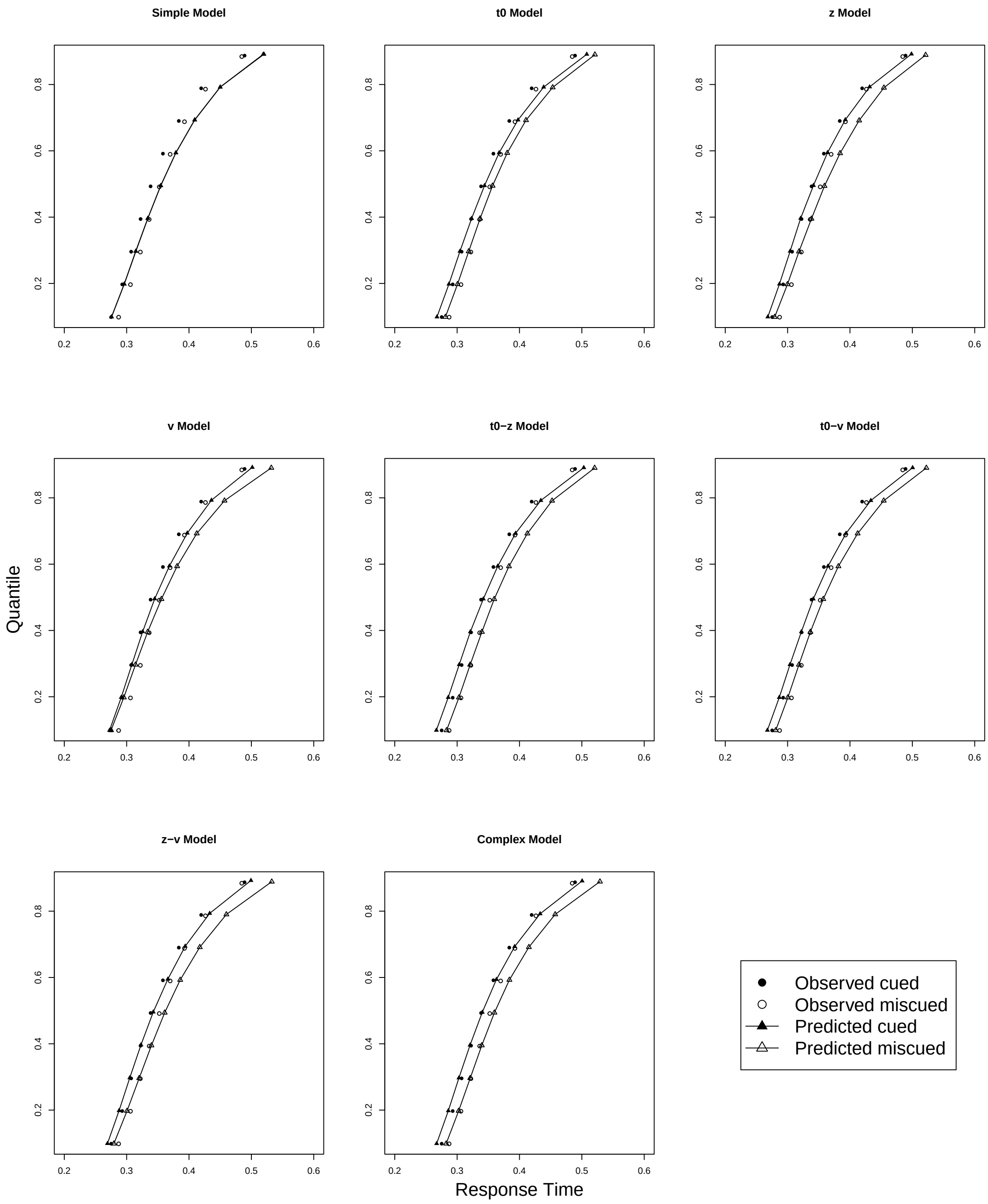

Supplementary Material 11: Qualitative Assessment of Moderating Factors in Data Set 3 with Between Trial Variability Parameters

As mentioned in the discussion, a limitation of our design was that we ignored several potential moderating factors in the original data sets. One of the main reasons why we did not assess these

Figure SM.12.

Data Set 2 Individual-Level Model Fits With Between Trial Variability Parameters

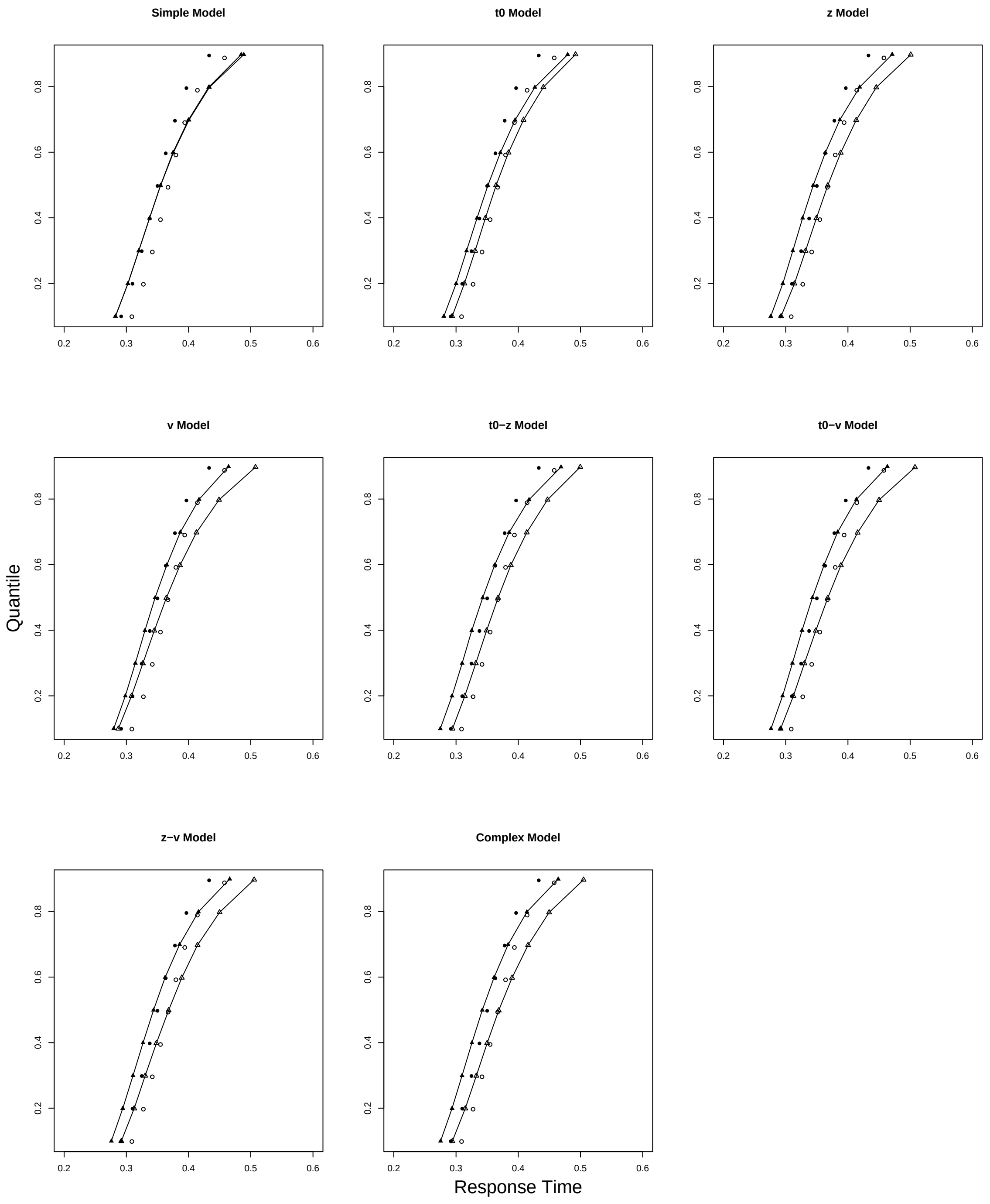

potential moderating factors was because in most cases, there were not enough trials to reliably estimate DDM parameters for these moderating factors. This was, however, possible for Data Set 3. In this supplementary material, we assessed each potential moderating factor: SOA (200ms, 500ms) and Cue Emotional Expression (Happy, Fearful, Neutral, Angry), as separate data sets to see whether our results changed as a function of these moderating factors.

Figure SM.13.

Data Set 3 Individual-Level Model Fits With Between Trial Variability Parameters

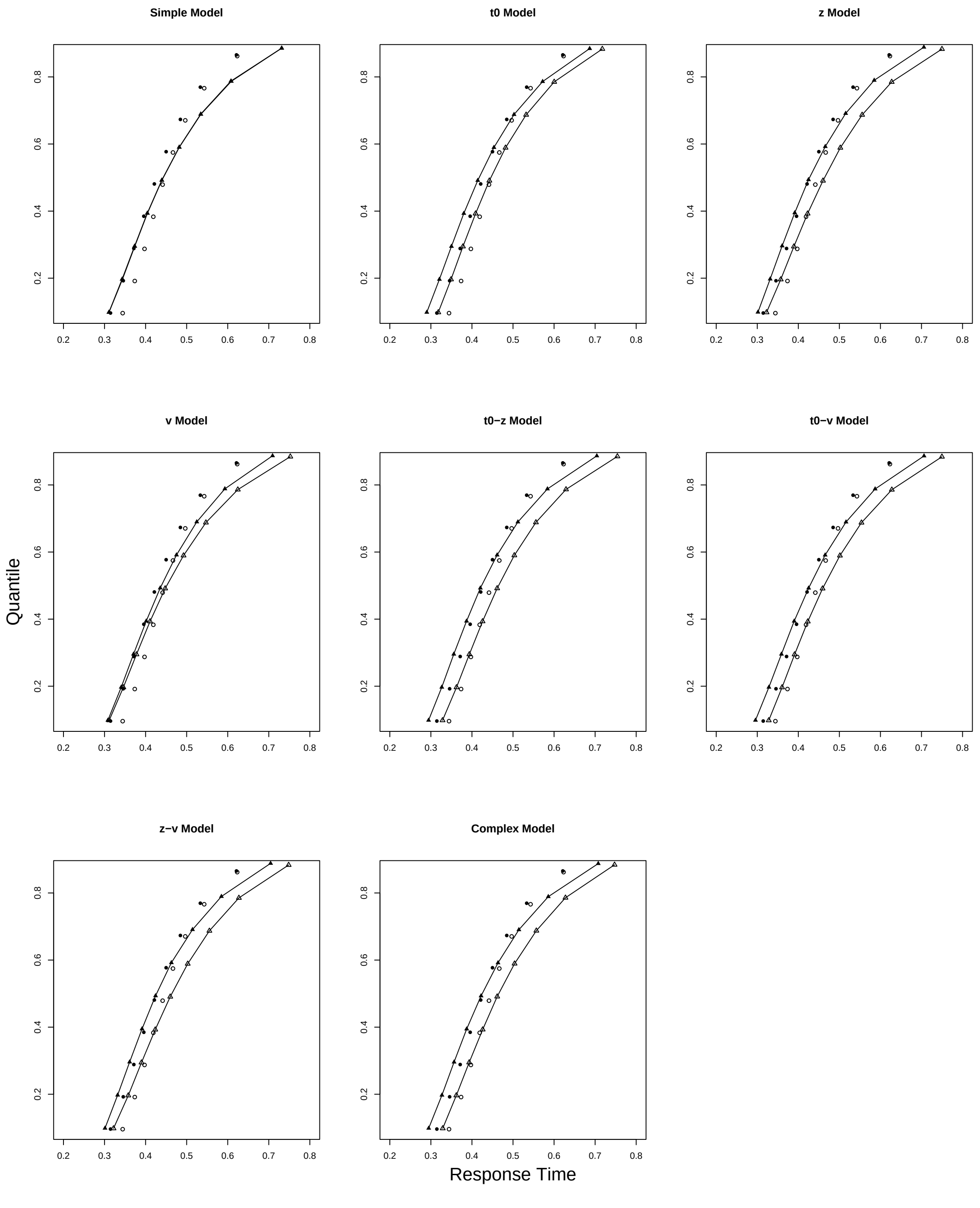

As shown in Figure SM.14 and Figure SM.15, there did not appear to be any obvious qualitative differences across moderating factors. We see a higher probability for the simple model in most plots, but this is because there were fewer trials and, particularly BIC, will favor simpler models when there are fewer trials. Therefore, we think it is reasonable to suggest that the overall conclusions from out paper hold across these SOA and cue emotion manipulations, at least for this data set.

Figure SM.14.

Weighted BIC Model Probabilities as a Function of Moderating Factor.

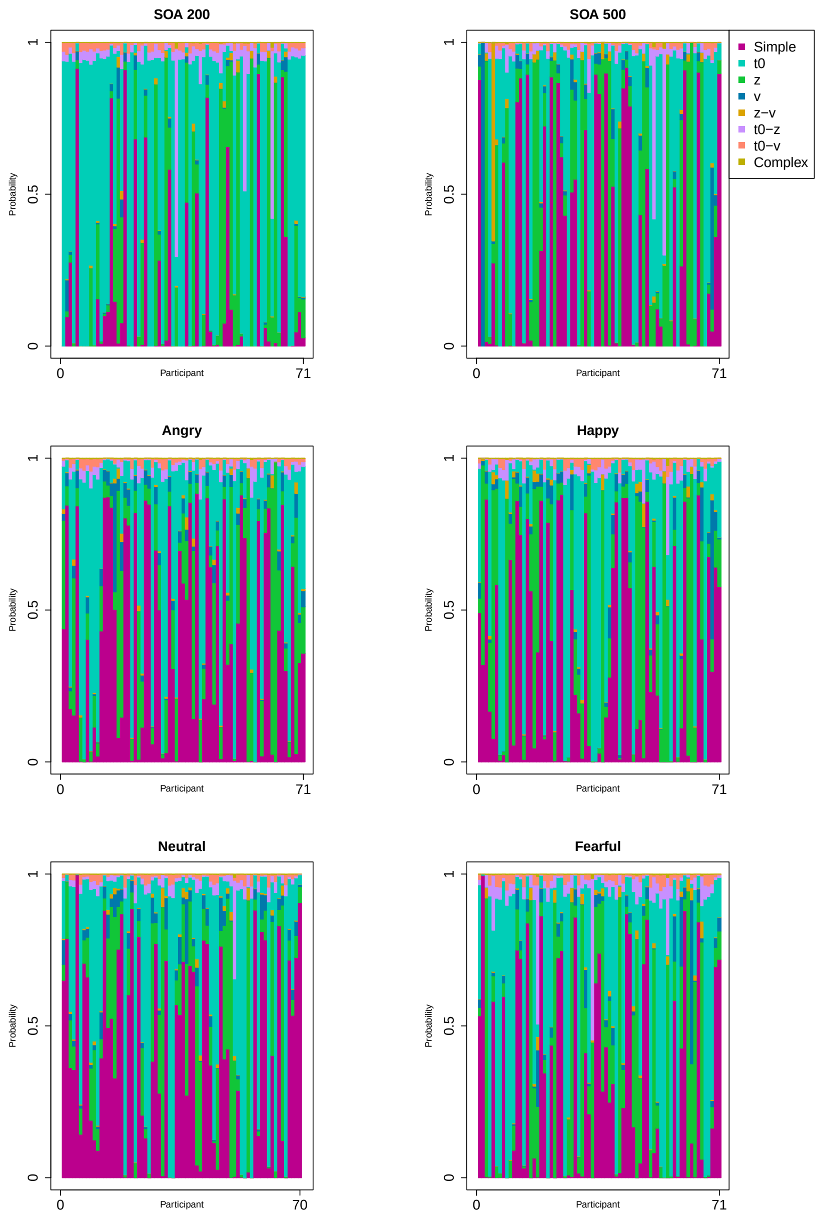

Supplementary Material 12: Arrow Cueing Analysis

Given the substantial research interest in comparing gaze-cues to arrow cues, we performed the same computational modelling analyses on an arrow cueing data set. We ultimately decided not to put these analyses in the main manuscript since we did not feel we had sufficient data to make any strong inferences, and did not want to detract from the main aim which was to understand gaze cues, but have included them here with the interest of prompting further research. We want to emphasise however, that any claims made about these data should be tentative, as there were relatively few trials per participant and few participants overall, particularly compared to the gaze

Figure SM.15.

Weighted BIC Model Probabilities as a Function of Moderating Factor.

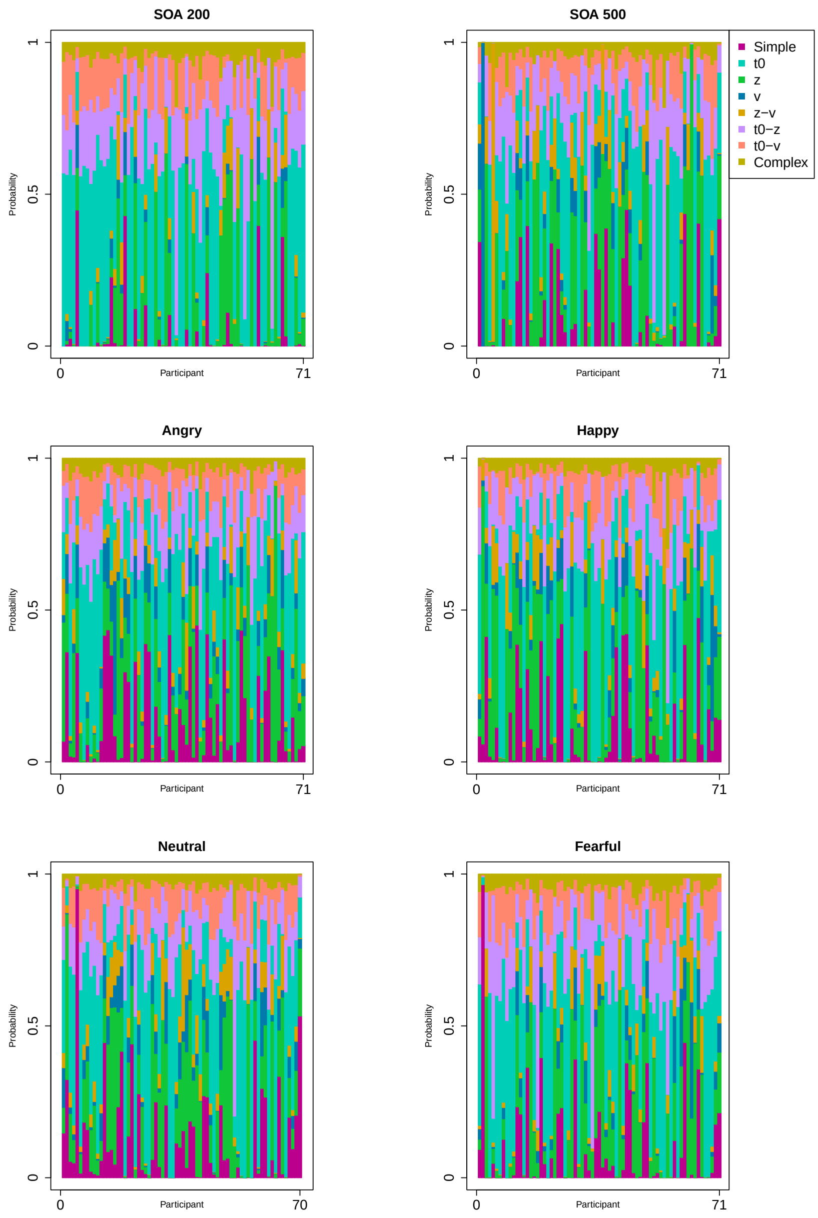

cueing data we analyse in the main manuscript.

Method

This data was from the same experiment, with the same participants as Data Set 1 in the main manuscript (Gregory & Jackson, 2020). The exact same exclusion criteria were applied. No participants were excluded, as they all had an accuracy of >80%. However, 21 trials (<0.01%) were removed from the arrow cueing data set for response times being <100ms or >5000ms, leaving a total of 9,819 arrow cueing trials analysed.

Results

Stage 1: Cueing Magnitudes

Thirty-nine out of the 41 participants displayed faster response times for cued trials compared to miscued trials on average, as shown in Figure SM.17. The mean cueing magnitude for the arrow cueing data including all 41 participants was 22ms (SD = 19; Standardized Mean Change = 0.53).

Figure SM.16. Cueing Magnitudes in Milliseconds for the Arrow Cueing Data Set

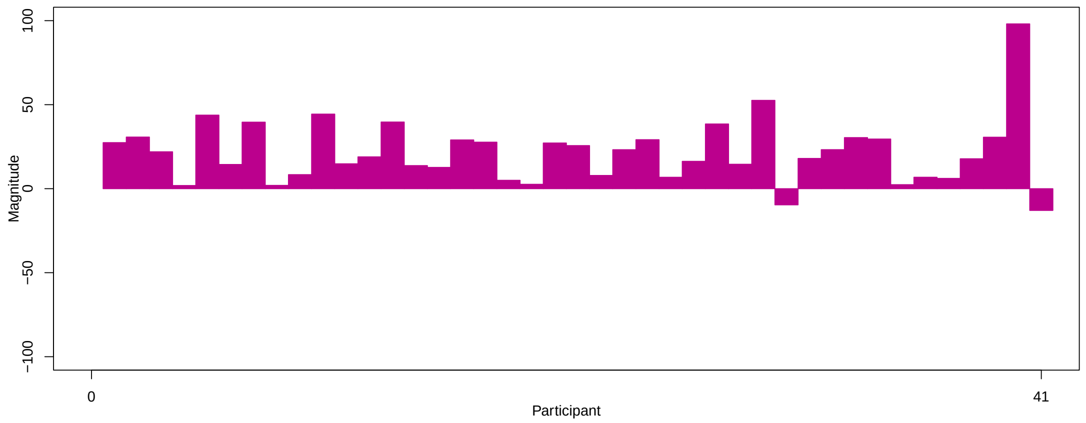

Note. Positive cueing magnitudes indicate faster mean responses to the cued location.

Stage 2: Individual-Level Modelling

Weighted Model Probabilities According to BIC, and as shown in Figure SM.16, 16 participants were best described by the simple model, 16 by the t0 model, 5 by the v model, 3 by the z model, and 1 by the t0-v model. When collapsing across participants and taking the mean, as shown in Table 6, the t0 model was the most likely to be the best performing candidate model followed by the simple model. According to AIC, 16 participants were best described by the t0 model, 11 by the simple model, 8 by the v model, 5 by the z model, and 1 by the v-t0 model. Averaging across participants, the t0 model was the most likely to be the best performing candidate model followed by the v and t0-v models respectively.

Parameter Inclusion Probabilities As shown in the second panel of Figure SM.18, 15 participants had greater than 50% chance of being best described by models that permitted t0 to vary across conditions. Two participants had greater than 50% chance of being best described by models that permitted z to vary across conditions. Five participants had greater than 50% chance of being best described by models that permitted v to vary across conditions.

Figure SM.17. BIC and AIC Weighted Model Probabilities for Each Participant

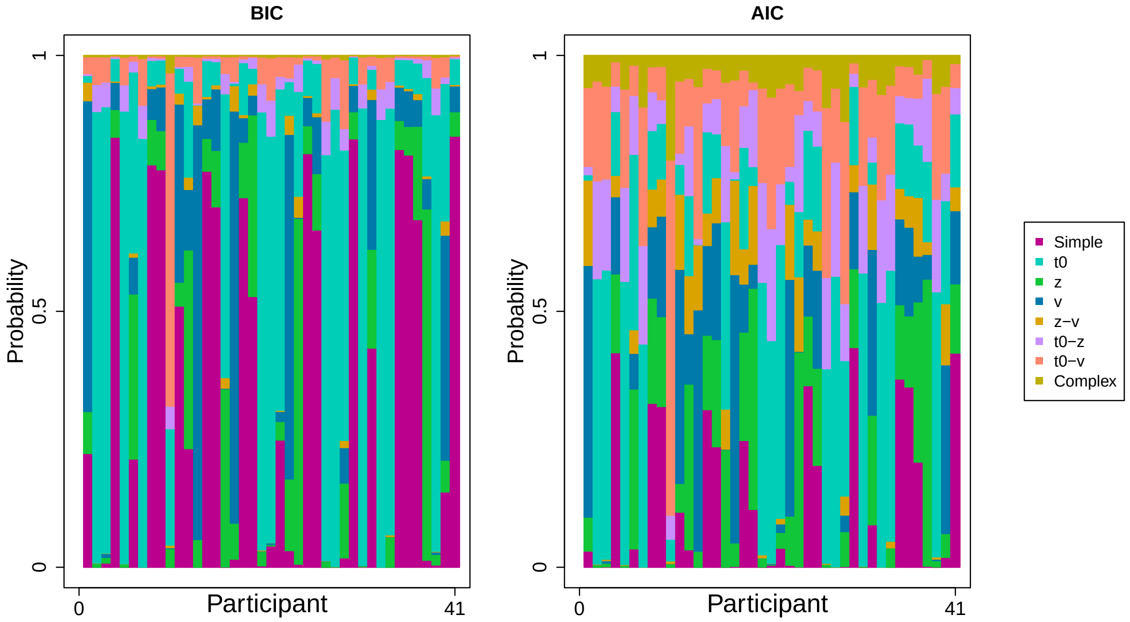

Collapsing across participants, models that assumed t0 varied across cued and miscued trials were on average 43% likely to be the best candidate model when compared to models that did not make this assumption (z, v, z-v, simple). Models that assumed z varied across cued and miscued trials were on average 16% likely to be the best candidate model compared to models that assumed z did not vary. Models that assumed v varied across cues and miscued trials were on average 20% likely to be the best candidate model compared to models that did not make this assumption. Therefore, as in Data Set 1a, according to these inclusion probabilities, t0 appeared to be the most likely out of the parameters of interest to be explaining any positive cueing effects in the observed data, although there was reasonable uncertainty.

At first glance these individual-level results would suggest that the arrow cueing effects and the gaze cueing effects in Data Set 1 were driven by common underlying mechanisms as they both showed the strongest evidence for an influence of t0 at the individual level. Interestingly however, inspection of Figures 6 and 7 in the main manuscript revealed that many of the people who had a high probability of being best described by t0 in Data Set arrow cueing were not the same people that had the highest probability of being best described by t0 in the gaze cueing (Data Set 1). Indeed, a post-hoc Bayesian correlation revealed that the there was moderate evidence for a lack of correlation between participants' t0 parameter inclusion probability in Data Set 1 and the arrow cueing data set (r = 0.04, BF = 0.36). Inspection of the cueing magnitudes in Figure 5 of the main manuscript and Figure SM.17 shows that this lack of consistency within participants maps onto their cueing magnitudes, whereby the same participants who had larger positive arrow

Table 6: Model performance as indicated by the individual-level modelling for arrow cueing collapsed across participants. "Probability" represents the relative probability of each model being the best candidate model according to BIC and AIC respectively. "Raw Score" corresponds to the raw BIC and AIC scores.

Model	BIC		AIC	
	Probability (%)	Raw Score	Probability (%)	Raw Score
Simple	31	-531.59	11	-542.07
t0	35	-537.66	25	-551.62
z	12	-535.09	13	-549.06
v	14	-532.38	14	-546.34
z-v	1	-530.21	6	-547.67
t0-v	5	-533.71	14	-551.16
t0-z	3	-533.16	11	-550.61
Complex	0	-528.44	5	-549.38

Note. Higher probabilities indicate a higher probability of that model being the best performing model. Lower raw BIC and AIC scores indicate better model performance.

cueing magnitudes tended to have smaller or negative gaze cueing cueing magnitudes in Data Set 1, statistically demonstrated by anecdotal evidence in favour of no correlation between cueing magnitudes in Data Set 1 and the arrow cueing data ($r = .20$, $BF = 0.70$). This lack of relationship between the gaze cues in Data Sets 1 and the arrow cueing data set suggests there may be differences across individuals in comparative responses to arrow and gaze cues. However, we would suggest that this may also be indicating that the data were noisy and/or unreliable. Ultimately, without more data it is difficult to know.

Stage 3: Bayesian Hierarchical Modelling

As shown in the second panel of Figure **??**, t0 had the most reliable difference in estimates across cued and miscued trials. Although this difference was not 95% credibly different from zero, the vast majority of the distribution suggested a parameter shift in the theoretically plausible direction and was closer to being 95% credibly different than zero compared to any of the other parameters. The mean difference in estimates for v was also in the theoretically plausible direction on average, however there was a lower probability that this estimate was reliably different from zero. The differences in estimates for z were the least likely to be shifting across conditions in the theoretically plausible direction.

Therefore, at the group level, these results suggest that t0 was the most likely to be driving any positive cueing effects at the group level, but that there is still considerable uncertainty. This uncertainty makes sense since the individual-level modelling found the simple model performed quite well for a large proportion of participants. In other words, it is plausible for any group-level

Figure SM.18. BIC Parameter Inclusion Probabilities for Each Participant and Data Set.

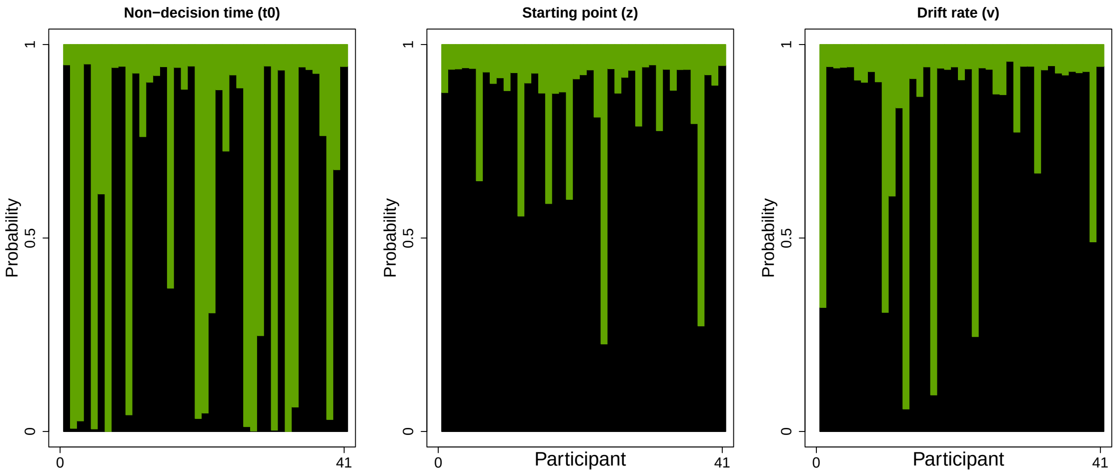

Note. Green represents models that assume the respective parameter varies across conditions. Larger proportion of green in these figures indicates broader support for a parameter driving differences across cued and miscued conditions.

Figure SM.19. Differences Between Hierarchical Bayesian Parameter Estimate Distributions Across Cued and Miscued Trials

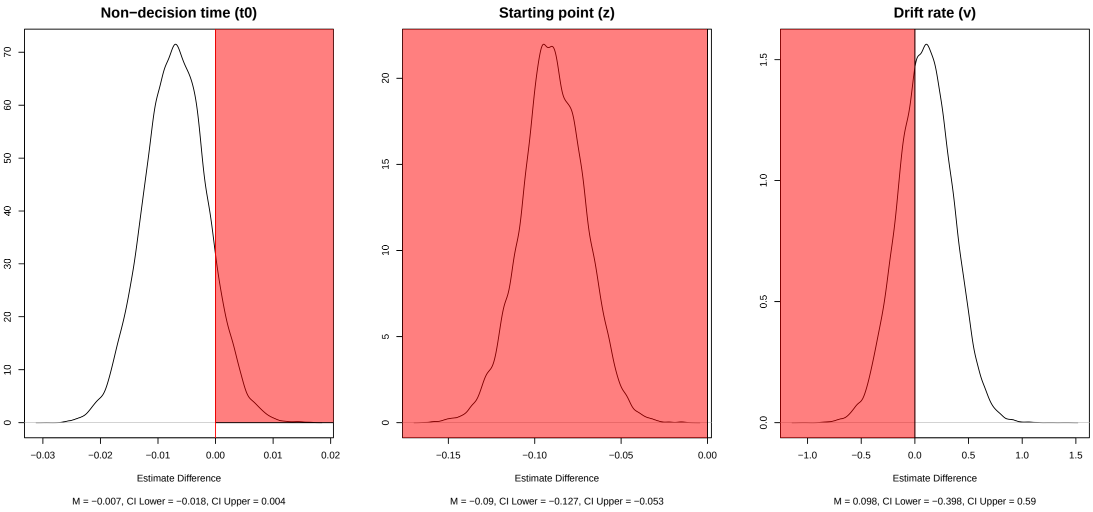

Note. The red portion of the plots represent the space of parameter differences which is inconsistent with the theoretically plausible direction for each parameter assuming a positive cueing effect. Specifically, in order to be considered theoretically plausible, t0 needed to show a negative difference across cued and miscued trials, whereas v and z needed to show positive differences. This plot shows a large proportion of differences close to zero and in the theoretically implausible direction, which we attribute to variability on the individual level.

effects to be highly uncertain when substantial individual variability is present. These group level results for arrow cues are similar to those uncovered for the gaze cues in Data Set 1, but with stronger evidence to suggest t0 is driving the effect, which is likely due to the fact that the cueing magnitudes tended to be larger for the arrow cues compared to Data Set 1 (see Figure SM.17 and

Figure 5 of the main manuscript).
